# Non-Invasive Blood Glucose Monitoring Using Machine Learning and Optical Approaches: A Systematic Literature Review and a Proposed Framework for Reproducible and Clinically Reliable Research

**DOI:** 10.3390/s26206572

**Published:** 2026-10-16

**Authors:** Syed Saim Ahmed, Norbert Noury

**Affiliations:** Institut for Nanotechnologies Lyon, University Claude Bernard, 69621 Lyon, France; syed-saim.ahmed@etu.univ-lyon1.fr

**Keywords:** blood glucose, diabetes mellitus, non-invasive monitoring, optical techniques, machine learning, shortcut learning, common framework

## Abstract

Diabetes Mellitus is the leading healthcare challenge. For effective management of this disease, patients need to check their blood glucose levels regularly; however, the standard method of taking a drop of blood and placing it on a test strip does not allow for continuous monitoring and is dreaded by most people, who find it painful and uncomfortable. Researchers worldwide are trying to develop non-invasive blood glucose monitoring (NIBGM) devices; most of them have reported promising accuracy using a combination of optical techniques and machine learning, but the CE/FDA marked solution still cannot reach the market. The main objective of this systematic review is to critically analyse recent quantitative research works in optical NIBGM, highlight their strengths, and identify the main factors that hinder regulatory approvals and the commercialisation of these devices. Web of Science, IEEE Xplore, PubMed, ScienceDirect, SPIE Digital Library, and Optica Publishing Group databases were searched between 1 September 2015 and 1 August 2026 using specific keywords. From the initial 1143 records, 245 studies were considered for full-text quality assessment, and 208 were excluded based on a post hoc quality score threshold. The sensing modality, wavelength, sample size, reference device, glucose range, validation strategy, accuracy metrics, and use of demographic variables as model inputs for the 37 included studies are reported. By applying an author-defined three-tier rubric, the risk of shortcut learning in each study was highlighted. Based on these findings, a common framework of fifteen concrete, evidence-grounded recommendations for future NIBGM studies was proposed.

## 1. Introduction

For the body to function normally, blood glucose levels should be between 80 and 150 mg/dl [1]. Higher or lower glucose levels, however, could cause several health problems. When food is broken down, glucose is produced. This molecule enters blood cells to provide energy and promote growth. The Insulin Hormone controls blood glucose levels by absorbing glucose from the blood and storing it as fuel for later use. Hyperglycaemia (high blood glucose concentration) and hypoglycaemia (low blood glucose concentration) are caused by abnormalities in insulin production and control. Diabetes Mellitus (DM) is a condition characterised by elevated blood glucose levels and physiological dysfunctions due to inadequate insulin production, insulin resistance, or excessive glucagon production [2]. A sudden increase in blood sugar level due to insufficient production of the insulin hormone is termed Type I Diabetes mellitus, while the unresponsiveness of body cells to the insulin hormone is known as Type II Diabetes Mellitus. Based on data from the International Diabetes Federation, 10.5% of individuals worldwide (about 536.6 million people) between the ages of 20 and 79 had diabetes in 2021. By 2045, that number is expected to rise to 783.2 million [3].

Diabetic people must check their blood glucose level regularly to avoid different complications such as stroke, cardiovascular disorders, tissue damage, kidney failure, etc. [4]. The majority of people around the world check their glucose level with the traditional invasive method, in which blood is drawn out of the body by puncturing the skin with a lancing device. Fifty percent of patients frequently hesitate to take their blood glucose level because of the expense, discomfort, and pain associated with the finger-pricking method [1]. Millions of people worldwide would benefit greatly from the development of a truly non-invasive glucose monitoring device [5]. NIBGM can help with continuous monitoring of health status by providing a much higher sampling rate, which is not achievable through invasive methods and thus has potential applications in wearable healthtech. Furthermore, by creating a Non-Invasive Device for Diabetic Management, significant volumes of medical and plastic waste produced by today’s invasive blood glucose Monitoring devices can also be avoided. Because of its huge market value and wide research gap: since 1991 [6,7] researchers have been trying to develop a Non-Invasive Glucometer; a few of them have also succeeded in developing commercial devices [8,9,10], but these devices are not commonly available to the people because of their high cost, low accuracy, and reliability as compared to traditional Invasive glucometers. Table 1 summarises notable techniques currently under research for non-invasive blood glucose monitoring.

Among all these approaches, optical techniques have proven more promising for non-invasive glucose monitoring due to their sensitivity and selectivity. Figure 1 indicates the penetration depth of various optical techniques.

However, despite significant progress in research, non-invasive, optically based blood glucose monitoring devices have still not reached the consumer market [26]. Due to the complexity of internal body structure and the sensitivity and selectivity limitations of optical techniques for NIBGM, there is now an increasing trend toward using artificial intelligence, particularly machine learning, for pattern recognition and prediction, as reported by Chan et al. in their scoping review [27]. Robert Geirhos et al. [28] first introduced the term “Short-Cut Learning” to the scientific community. Shortcuts are decision rules that perform well on both training and independently and identically distributed (i.i.d.) test data, yet rely on unintended, superficial correlations present in the dataset rather than on the intended, causally relevant features and they fail systematically when evaluated on out-of-distribution (o.o.d.) data. Recently, Hui Jiang et al. [29] reviewed 106 peer-reviewed articles on photoplethysmography-based blood glucose monitoring. They reported that the devices fail to maintain accuracy in populations with diverse physiological and anatomical variance. After conducting a scoping review using the Arksey and O’Malley five-step framework, Chan et al. explicitly highlighted the need to develop consensus guidelines and protocols to ensure the quality and safety of AI-enabled non-invasive blood glucose monitoring systems.

To address this scientific need, the main goal of our work was to critically analyse recent state-of-the-art research on AI-enabled optical approaches for NIBGM, to highlight the challenges and opportunities for researchers, and to propose a common research framework. This framework would allow experimental results to be compared under the same conditions across research groups, enabling the design and development of robust, accurate, and reliable devices for effective diabetes management.

Our main research question was as follows: “In optical or hybrid non-invasive blood glucose monitoring systems, how do different signal acquisition, signal processing techniques, and AI algorithms, compared with invasive glucose measurements, affect the accuracy, reliability, and robustness of glucose predictions across pre-clinical and clinical studies?”

The question can be broken down into the following sub-questions: (i) “Which are the main types of optical NIBGM methods used in literature studies?”; (ii) “What types of instrumentation, wavelengths, and signal processing were adopted in the literature studies?”; (iii) “Which experimental protocols were performed for data acquisition, and how representative were the study populations of the diabetic population?”; (iv) “Which algorithms are used in the scientific literature for the estimation of blood glucose?”; and (v) “How was the validation of optical NIBGM systems performed?”.

## 2. Materials and Methods

This systematic review was conducted and is reported in accordance with the PRISMA 2020 statement [30].

### 2.1. Information Sources and Search Strategy

We searched the Web of Science, PubMed, ScienceDirect, and IEEE Xplore databases using the Boolean strategy shown in Table 2. To fill a gap in optics-specific literature, we also searched SPIE Digital Library and the Optica Publishing Group directly.

### 2.2. Study Selection

We imported all retrieved citations into EndNote and removed duplicates. Two reviewers (S.S.A. and N.N.) then independently screened titles and abstracts against the eligibility criteria. A record was excluded at this stage only if both reviewers agreed it clearly did not meet the criteria. Any disagreements were resolved through discussion, and if consensus could not be reached, the record moved on to full-text assessment.

### 2.3. Eligibility Criteria

- The study reports an optical or hybrid optical + non-optical technique applied specifically to non-invasive blood glucose estimation.
- The study reports at least one machine-learning, deep-learning, or comparable mathematical algorithm used for glucose estimation from the acquired signal.
- The study is published as a peer-reviewed journal article or as a peer-reviewed conference proceedings paper. Conference papers are included because a substantial proportion of engineering and biomedical-optics work in this fast-moving field is first, and sometimes only, published in venues such as IEEE EMBC, IEEE Sensors, and SPIE/Optica proceedings, among others.
- The study reports at least one quantitative performance metric (e.g., MAE, RMSE, MARD, Clarke/Parkes Error Grid zone distribution, classification accuracy, or correlation coefficient) against a defined reference measurement.
- The study was published in English between September 2015 and August 2026.
- The study is not a review, editorial, or conference abstract without a full methods and results section.

### 2.4. Data Extraction

For each study that passed title and abstract screening, we recorded the sensing technique, the wavelength or physical principle, the machine learning or deep learning method used, the number of participants, the reference device for true blood glucose measurements, and the reported accuracy.

### 2.5. Quality Assessment

The 245 studies that passed title and abstract screening still differed a lot in methodological quality. To assess them consistently, we scored the full text of each study across seven domains: publication venue quality, instrumentation and data collection, study population, reference standard and performance reporting, validation methodology, machine learning/deep learning rigour, and confounder transparency. Studies could receive up to 100 points in total. The full scoring rubric, with points assigned at each level of every domain, is provided in Supplementary Table S1. This seven-domain scoring rubric was developed by the review authors for this review; it is an author-defined assessment tool rather than a validated or externally standardised instrument, and the 37 included studies were therefore selected according to author-defined criteria.

Studies scoring at least 63 out of 100 were included in the review. The threshold was chosen after we examined the distribution of scores across all assessed studies, in which the lowest-scoring included study scored 63, and the highest-scoring excluded study scored 60. Because it is a post-hoc choice, it may introduce selection bias. A sensitivity analysis using cut-offs of 58 and 68 is reported in Section 4.5.

### 2.6. Study Selection Results

Figure 2 summarises the complete study-selection process. We initially retrieved 1143 records from six databases. After removing duplicates, 140 within ScienceDirect and 79 across the other databases, 924 unique records remained. During title and abstract screening, we excluded 679 records that were review articles, unrelated to glucose, did not use optical sensing, did not apply machine learning or deep learning methods, or reported only in vitro or simulation results without human data. This left 245 records for full-text quality assessment, as described above. After the final assessment and discussion among authors, 37 papers were included in the review.

## 3. Results

### 3.1. Overview of Sensing Modalities

Photoplethysmography (PPG), used alone or combined with a second physiological channel, was the most common sensing approach, used in 18 of 37 studies (49%) [31,32,33,34,35,36,37,38,39,40,41,42,43,44,45,46,47,48]. Figure 3 summarises the sensing-modality distribution across all 37 included studies. Two of the PPG-based studies used the publicly available Mazandaran PPG dataset (67 raw PPG signals from 23 volunteers) [49]. Şentürk et al. [35] used it for training and testing, and Chellamani et al. [42], who refer to it as MUST, used it only for testing. Near- and mid-infrared (NIR/MIR) absorption spectroscopy was the second most common approach, used in 9 studies [50,51,52,53,54,55,56,57,58], followed by Raman spectroscopy in 5 studies [59,60,61,62,63]. Photoacoustic spectroscopy was used in 2 studies [64,65], while optical coherence tomography, polarimetry, and hybrid millimeter-wave/NIR sensing were each used in a single study [66,67,68]. This distribution likely reflects PPG’s low hardware cost and compatibility with existing consumer wearables, rather than necessarily indicating which modality has the strongest physical basis for glucose specificity.

### 3.2. Wavelengths Used in PPG and NIR/MIR Studies

Two wavelength clusters dominate this literature, shown in Figure 4. The first is around 850–950 nm, which overlaps with the classic pulse-oximetry infrared band. The second is around 500–700 nm, covering the green/red visible range used for reflective PPG sensing. A third, smaller cluster spans 1200–1650 nm. This region corresponds to glucose-specific overtone and combination absorption bands, and is targeted by more spectroscopically motivated designs [38,52,53,57,58]. This pattern is informative. Studies using wavelengths near the pulse-oximetry bands are, based on the physics at those wavelengths, more likely to measure haemodynamic correlates of glucose such as blood volume and vascular tone, rather than glucose’s own weak overtone absorption. In contrast, the 1200–1650 nm cluster lies much closer to wavelengths with a direct, established physical link to glucose absorption as mentioned by Taixiang Li et al. [38].

### 3.3. Wavelengths Used in Raman Spectroscopy Studies

All 5 Raman studies, shown in Figure 5, use near-infrared excitation between 785 and 830 nm [59,60,61,62,63], a narrow, deliberately chosen range that minimises tissue autofluorescence while remaining within a wavelength band where silicon-based CCD detectors remain sensitive.

### 3.4. Wavelengths Used in OCT and Photoacoustic Studies

Both photoacoustic studies, shown in Figure 6, used an identical dual-wavelength setup at 905 nm and 1550 nm [64,65]. By targeting different absorption properties at each wavelength, they aimed to distinguish glucose signals from background tissue absorption. The one OCT study used a 1310 nm swept-source laser [66]. This wavelength is common in telecommunications and was selected for its strong tissue penetration and reduced scattering, not for direct glucose absorption.

### 3.5. Reference Blood Glucose Measurement Method

Figure 7 shows the wide variety of devices used to measure reference blood glucose levels. Venous blood drawn and analysed by a clinical laboratory was the most common reference standard, used in nine studies (24%) [37,42,46,48,52,53,61,62,68]. Five studies did not report any reference device at all [32,35,47,54,58]. Continuous glucose monitors (CGMs), mostly the Dexcom G6, were used in four studies [36,40,45,57]. Accu-Chek glucometers appeared in nine studies, but across there were different models: Instant, Performa, Guide, and unspecified [33,38,41,43,44,51,60,63,64]. This means that even within the same manufacturer, the specific reference device varied from study to study. The remaining studies used other commercial glucometers including CareSens, VivaChek, CareTouch, HemoCue, OneTouch, Yuwell, FreeStyle Libre, and Contour Next One.

### 3.6. Sample Size (N) Distribution

The majority of studies, 27 of 37 (73%), enrolled fewer than 100 participants, as shown in Figure 8. Several included fewer than 10 [43,54,56,57,68]. Only four studies (11%) enrolled more than 250 participants [37,38,44,47]. This strong bias toward small cohorts is important for the shortcut-learning assessment as mentioned in the discussion section.

### 3.7. Reference Blood Glucose Range Validated

Fourteen studies (38%), shown in Figure 9, validated across the 70–250 mg/dL range, the most common band. This covers normal to moderate hyperglycaemic values [31,32,35,39,41,43,44,52,53,57,59,63,64,65]. Thirteen studies (35%) tested across a wider range extending beyond 250 mg/dL [33,40,42,47,50,51,55,58,61,62,66,67,68]. Of these, nine studies specifically included confirmed hypoglycaemic readings below 70 mg/dL [40,42,50,53,55,61,62,67,68]. Only four studies (11%) were limited to a narrow 70–150 mg/dL band [34,37,46,56], and six studies (16%) did not report a clear numeric glucose range [36,38,45,48,54,60].

### 3.8. Accuracy Metrics Reported

Most studies, shown in Figure 10, reported more than one metric (29 of 37, 78%). The most common combination was a point-error metric such as MAE, MARD, or RMSE together with a Clarke (most cases) or Parkes Error Grid analysis. Four studies (11%) reported only RMSE, with no point-error or error-grid metric [52,53,54,59]. Another four studies (11%) reported only Clarke Error Grid distribution-based results clearly.

### 3.9. Validation Method

A simple hold-out (train/test) split, shown in Figure 11, was the most commonly reported validation method, used in 19 studies (51%) [31,32,34,35,42,43,44,48,51,53,54,57,58,59,60,62,63,64,65]. This was followed by k-fold cross-validation in 10 studies (27%) [36,37,38,39,40,41,45,46,56,61] and leave-one-out cross-validation in four studies [50,52,55,67]. Four studies (11%) did not report which of these three split types (hold-out, k-fold, or leave-one-out) was used [33,47,66,68]; this is a separate question from whether the split was subject-wise, which is assessed independently for the shortcut-learning rubric (Section 3.14). For example, Kusumo et al. [47] did not state which split type was used, but did report explicit subject-level data partitioning. Critically, the validation method label alone does not indicate whether a split was subject-wise, (with test participants entirely unseen during training), or sample-wise, (with segments from the same participant potentially appearing in both the training and test sets).

### 3.10. Units of Reported Accuracy

Twenty-five studies (68%), shown in Figure 12, reported results in mg/dL, while twelve studies (32%) used mmol/L. This split makes it difficult for readers to compare accuracy figures directly across studies without converting units (1 mmol/L = 18.0 mg/dL).

### 3.11. Deep Learning vs. Simple/Classical Machine Learning

Simple or classical machine-learning methods, shown in Figure 13, including regression, tree ensembles, support vector machines, and similar approaches, remain more common than deep-learning architectures. Deep-learning based approaches are prominent in PPG based NIBGM. While in NIR, Raman, photoacoustic, OCT, and polarimetry-based studies, classical regression-based approaches clearly dominates [50,51,52,53,55,56,59,60,61,63].

### 3.12. Feature Set Used for Model Training

The large majority of studies, shown in Figure 14, trained their predictive model on signal-derived features only, with no demographic or anthropometric variables included as direct model inputs. Seven studies explicitly included demographic or anthropometric variables in the model alongside signal-derived features: BMI [32,64]; demographics combined with temperature and humidity [38]; age [48]; a full demographic panel including age, sex, BMI, meal status, diabetic status, sleep, stress, and SpO2 [51]; BMI as part of a final calibration-success model [55]; and sex, weight, and height fed directly into a BiLSTM [58].

### 3.13. Real-Time or Prospective Testing on New Data

Only two of thirty-seven studies, shown in Figure 15, reported any form of real-time or prospective testing beyond offline train/test evaluation [46,47]. In both cases, reporting was explicitly incomplete.

Ahuja et al. [46] described a real-time deployment test but provided limited detail on subject characteristics. They also mentioned that accuracy degraded from R^2^ = 0.989 offline to R^2^ = 0.984 in deployment. Kusumo et al. [47] stated that real-time testing was performed but did not report the results with enough detail to assess external validity. Rothenbühler et al. [62] describe a "prospective pilot study" in its title, referring to its prospective cohort-collection design; the study itself reports no real-time or post-deployment testing of a finalised model, and is not counted here. So, the remaining 35 studies (95%) reported no real-time testing at all.

The near-total absence of real-time, prospective validation means that essentially all reported accuracy figures describe offline, retrospective model performance. This is a materially different, and generally more favourable, evaluation of condition than the real-world deployment.

### 3.14. Shortcut-Learning Risk Classification

Each of the 37 studies was rated as Low, Medium, or High risk using an author-defined rubric applied to the methods reported in each study, rather than to the reported accuracy values, summarised in Figure 16. A study was classified as High risk if at least one of three criteria applied: (i) a demographic or anthropometric variable (e.g., BMI, age, sex) was used as a direct input to the predictive model; (ii) the study reported accuracy from a very small cohort, operationally defined as fewer than 10 participants, together with near-perfect accuracy, operationally defined as MAE of less than 5 mg/dL, without a confirmed external test subject-wise; or (iii) the study’s own authors reported evidence of memorisation, overfitting, or a substantial accuracy drop on unseen participants. A study was classified as Medium risk if subject-wise validation could not be confirmed, or if a personalised per-subject model was used, but none of the three High-risk criteria applied. A study was classified as Low risk if it confirmed subject-wise validation, avoided demographic model inputs, and provided evidence that the optical signal itself, rather than a confounding factor, was driving accuracy. The choice of validation strategy (Section 3.9) and whether prospective or real-time testing was reported (Section 3.13) are tracked and discussed separately throughout this review; on their own, neither an unconfirmed subject-wise split nor the absence of real-time testing is sufficient for a High rating under this rubric, although both reduce confidence in the reported accuracy of any study, regardless of its risk tier.

The shortcut-learning risk ratings were assigned by one reviewer (S.S.A.), who read the full text of each included study and rated it Low, Medium, or High according to the rubrics mentioned and provided in the Supplementary Table. A second reviewer (N.N.) then checked every rating against the original paper and the rubric criteria to confirm that the evidence recorded was accurately reported and that the rubric had been applied consistently. Where the second reviewer disagreed with a rating or the supporting evidence, the two reviewers discussed the study and agreed on a final rating by consensus. Because the second reviewer verified existing ratings rather than rating each study separately, the two assessments were not independent, and no inter-rater agreement statistic could be calculated.

Applying this rubric, 23 of 37 studies (62%) were classified as High risk [31,32,35,38,39,40,41,42,43,44,45,48,51,52,53,54,55,56,57,58,60,64,68], 8 (22%) as Medium [33,34,36,46,50,65,66,67], and 6 (16%) as Low [37,47,59,61,62,63].

## 4. Discussion

The normal blood glucose level of a person is between 70 and 150 mg/dL [69,70]. If we assume that the density of water is one, then there will be 78 g to 80 g of water in one deciliter of blood [71]. In normal subjects, the overall mean haemoglobin concentration is 13.60 g [72]. It implies that water and red blood cells create major interference while measuring blood glucose levels non-invasively using an optical method. Yu Shimojo et al. proved that the light absorption coefficient of epidermis, dermis, and subcutaneous fat varies significantly by performing experiments using light of a 400 to 1100 nm wavelength range [73]. L. Souza-Barros et al. [74] demonstrated the direct effect of melanin (responsible for skin colour) on optical transmission in body tissues. Hannah D. R. Gruensfelder et al. [75] explained the effect of pigmentation (skin colour) on the readings of optical instruments, and they highlighted that water, melanin, oxyhaemoglobin, fats, and deoxyhaemoglobin are the main interfering agents in NIBGM.

### 4.1. Risks Inherent in the Currently Published NIBGM Literature

The wavelength analysis in Section 3.2 shows that studies clustering near the classical pulse-oximetry bands (660/940 nm) are, based on underlying physics, more likely to be sensing haemodynamic correlates of glucose such as blood volume and vascular tone, rather than glucose’s own weak overtone absorption. This is the same physical limitation described by Jiang et al. [29] in their recent review of PPG-based glucose sensors, and it is verified here by the fact that only the smaller cluster of studies [38,52,53,57,58] deliberately targeted wavelengths of 1200–1650 nm, which have an established direct physical link to glucose absorption in terms of sensitivity and specificity. The correlating factors vary from person to person, which is why the majority of machine learning and PPG-based blood glucose monitoring devices fail during real-time application in more diverse populations. All five studies in the 1200–1650 nm cluster [38,52,53,57,58] were rated High risk (Section 3.14) because of factors such as a demographic variable used as a direct model input, criterion (i) [38,58]; a small cohort combined with near-perfect accuracy, criterion (ii) [52]; or the study’s own authors reporting a substantial accuracy drop on unseen participants, criterion (iii) [57].

A second, closely related risk concerns the direct use of demographic and anthropometric variables as trained-model inputs. Seven of the 37 studies [32,38,48,51,55,58,64] include a demographic variable, most commonly BMI or age, directly in the feature set used to train the predictive model, rather than reporting it only as a covariate. In at least one of these, Sen Gupta et al. [32], the original authors themselves report that BMI was more strongly correlated with the training labels than any feature extracted from the optical signal, which is an indication that the model’s apparent accuracy may not be attributable to the optical signal at all.

A third risk concerns sample size relative to model capacity. Five of the 37 studies [43,54,56,57,68] reported results from fewer than 10 participants, and 27 of 37 (73%) reported results from fewer than 100 participants. A high-capacity model, whether a deep neural network or a classical regressor with many engineered features, that is trained and evaluated on a cohort of this size has considerably more opportunity to fit person-specific signal characteristics than to learn a genuinely generalisable optical-glucose relationship. This risk is not merely theoretical. For example, on a sample of just four participants, Song et al. [56] reported average R^2^ = 0.97 and 100% in Zone A. Chuchkanov and Velyhotskyi et al. [43] reported an RMSE of 0.378 mmol/L (about 6.8 mg/dL) from data collected from a single healthy participant. Similarly, Kim et al. [57] provided direct support for this concern by reporting that a deep neural network trained data from five subjects achieved an RMSE of 14.13 mg/dL, but the error increased to 25–69 mg/dL when tested on unseen subjects.

Another issue is the near-total absence of real-time or prospective validation. Only 2 of the 37 studies [46,47] report any testing beyond offline train/test evaluation, and in both cases the reporting is incomplete. Ahuja et al. [46] report a measurable accuracy drop from R^2^ = 0.989 offline to R^2^ = 0.984 during deployment but provide limited subject-level detail. Kusumo et al. [47] state that real-time testing was performed, but do not report results in sufficient detail to assess external validity. The remaining 35 studies report no real-time testing at all. This means that the majority of the literature describes offline model performance, which represents a materially more favourable evaluation condition than real-world deployment. In deployment, factors such as motion artefacts, ambient temperature, sensor repositioning, and genuinely unseen physiology due to vasodilation and vasoconstriction are present simultaneously.

These factors are consistent with a high risk of shortcut learning in a majority of the optical blood glucose monitoring systems reviewed here, under our screening rubric. We do not claim that this review identifies the cause of any specific device’s failure; rather, it documents methodological weaknesses that would possibly reduce the robustness once a model is evaluated on a population where the learned correlation no longer holds. For example, this could occur in an older or younger cohort with a different baseline glucose-BMI relationship or skin tone.

Studies based on Raman spectroscopy carry, on average, a lower risk of shortcut learning than other modalities, owing to a clear, glucose-specific spectral signature. Zhang et al. [61] and Rothenbühler et al. [62], both rated Low or Medium risk, reported the highest sensitivity and specificity for glucose within the 1100–1150 cm^−1^ Raman wavenumber band. However, the study conducted by Liu et al. [60] combined data from animal models and human subjects, and the authors themselves reported that accuracy was stable when the model was fitted to a single subject, but declined in multi-subject testing due to inter-sample variance. This was due to limited generalisation, not a weakness in Raman spectroscopy itself (Section 3.14).

A model trained on physiological or demographic variables such as BMI, age, or skin tone may primarily learn population-level demographic correlations rather than a genuine glucose-dependent optical signature. This distinction matters because a demographic correlation is not stable across populations: it can differ by age group, sex, ethnicity, or skin pigmentation, and a model that relies on it will not transfer reliably to a population where the correlation does not hold in the same way. This does not mean demographic variables have no place in NIBGM research. They remain valuable for confounder control, subgroup analysis, calibration-transfer assessment, and fairness evaluation across skin tones and body types. The distinction we draw is between recording a demographic variable for these analytical purposes and using it as a direct input feature to the predictive model itself.

Hold-out and k-fold cross-validation are the most commonly reported validation strategies in this literature (Section 3.9), but neither guarantees that a reported accuracy figure will generalise to a new patient. If multiple signal segments, or repeated measurements, from the same participant are distributed across both the training and test folds, a validation split at the sample level allows the model to partially memorise that participant’s individual signal characteristics rather than learn a relationship that holds across participants, as shown in comparable clinical machine-learning settings [76]. For this reason, subject-wise splitting, in which all data from a given participant is assigned entirely to one fold, and independent external validation on a separate participant cohort, are both necessary to assess whether a model has learned a generalisable signal rather than a memorised one.

### 4.2. Why a Common Framework Is Needed?

Section 3.11 shows that classical machine learning and deep learning are used with almost equal frequency in the PPG literature, while classical regression clearly dominates the NIR, Raman, photoacoustic, OCT, and polarimetry literature [50,51,52,53,55,56,59,60,61,63]. For traditional machine learning algorithms, features need to be extracted, and only statistical features from optical sensors are not sufficient for accurate NIBGM due to the interference factors described above. There is a need to extract more diverse and significant features in the time, frequency, and wavelet domains to uncover the hidden patterns in optical signals.

Data is the input of ML models and thus plays an integral role in their performance. Poor and limited data will lead to poor prediction under real-world conditions, that is, a lack of generalisation [43], and Section 3.6 shows that nearly three-quarters of the 37 studies enrolled fewer than 100 participants. Many of the studies reviewed here collected data predominantly from participants having normal glycaemic range and, on this basis, concluded that their device would work for diabetic management; however, the main intended users of NIBGM devices are diabetic patients, whose glucose levels can exceed 300 mg/dL, and the accuracy of a device validated only in the normal range cannot be assumed to hold at these extremes. Out of the 37 studies, 9 reported validation extending into confirmed hypoglycaemic readings below 70 mg/dL [40,42,50,53,55,61,62,67,68], but 6 studies still do not report a numeric glucose range at all [36,38,45,48,54,60], and 4 studies remain confined to a narrow, near-normoglycaemic 70–150 mg/dL band [34,37,46,56].

The training and evaluation of a machine learning model are crucial when designing intelligent medical devices. Many researchers prefer hold-out and k-fold cross-validation approaches [44]. Section 3.9 shows that a simple hold-out split remains the single most common approach, used in 19 of 37 studies [31,32,34,35,42,43,44,48,51,53,54,57,58,59,60,62,63,64,65]. This is followed by k-fold cross-validation in 10 studies (27%) and leave-one-out cross-validation in 4 studies. Four studies did not report a clear validation methodology at all. Critically, the validation-method label alone does not indicate whether a split was subject-wise, with test participants entirely unseen during training, or merely sample-wise, with segments from the same participant potentially appearing in both the training and test sets. This distinction determines whether a reported accuracy figure reflects generalisable sensing or partial memorisation of the training cohort.

Figure 7 shows the wide variety of devices used to measure reference blood glucose levels. Glucose concentration is not uniform across the body: measurable differences exist between venous blood, capillary blood, and interstitial fluid, especially after a glucose load rather than in the fasting state [77]. Furthermore, glucometers have inherent measurement tolerance both across manufacturers and across different models from the same manufacturer. The ISO 15197:2013 standard considers a blood glucose meter acceptably accurate if at least 95% of its results fall within ±15 mg/dL (0.83 mmol/L), or ±15% for higher concentrations, of the reference method’s result [78]. ISO 15197:2013 was formally adopted in Europe as EN ISO 15197:2015; the two refer to the same accuracy requirements [79]. Even among devices that meet this standard, accuracy evaluations of CE-marked systems have found considerable variation in performance, with the smallest acceptable deviation across tested meters being ±7.7 mg/dL and the largest reaching ±19.7 mg/dL. This combination of physiological variability across sample types and the permitted and observed measurement tolerance across devices makes it difficult to treat readings from different devices directly as interchangeable. It also complicates the integration of previously collected data with the new data, particularly since a specific device model may not be available in every geographical location.

Due to legal and ethical restrictions, data collection remains a major challenge for researchers, and reliable, publicly available datasets for non-invasive blood glucose monitoring remain scarce. It would benefit the scientific community to establish and follow a common framework for investigation: proper protocols for the minimum number of participants, the percentage of diabetic patients, different skin colour types, and a minimum duration for acquiring optical signals must be considered, and datasets should be shared so that better algorithms can be designed to improve accuracy.

### 4.3. Proposed Common Framework

Based on the evidence presented in Section 3 and Section 4.1, we propose a common framework for NIBGM research. Each recommendation below is stated as a methodological criterion for evaluating the performance and generalisability of a machine-learning model trained to predict NIBGM from optical signals.

For visible and infrared spectroscopy based on absorption and scattering principles, the minimum duration of collected optical signals should be at least 20 s, so that information below 0.5 Hz can also be properly analysed. This recommendation was made on the basis that currently no specific frequency range has been identified where the exact information of blood glucose level lies, so instead of eliminating low-frequency-based signals, we should analyse them properly and report results accordingly. Normally, the higher cut-off frequency while capturing PPG signals is kept at 14 Hz; to reduce aliasing effects, as per the Nyquist theorem [80], the sampling frequency should be greater than 28 Hz. It applies only to the PPG signal, not in general to all other optical techniques. For every other case, researchers need to ensure that the sampling frequency is at least twice the maximum frequency of interest.

The best reference for calibration and labelling is still an invasive capillary glucometer or a lab-based venous plasma test. There are different options available, and researchers can choose as per their convenience. However, to make results reproducible and easy to compare, it would be better to use a common reference device, e.g., Roche Accu-Chek Guide Glucometer, as Roche distributes them in more than 100 countries [81], so most research groups can access the same device. Independent testing shows that Accu-Chek Guide strips exceed the ISO 15197:2013 standard. They meet a stricter "10/10" accuracy level instead of just the minimum "15/15" requirement [82]. For continuous reference measurements, similarly Abbott’s FreeStyle Libre 3 can be used. It is available in over 40 countries [83] and has a manufacturer-reported mean absolute relative difference of 9.2% in adults [84].

For participant recruitment, we recommend that studies include enough people to match the complexity of the model being trained. More importantly for NIBGM, our review found that 27 of 37 studies (73%) used fewer than 100 participants. Small sample size, combined with near-perfect reported accuracy, is one of the three rubric criteria that can produce a High shortcut-learning-risk rating (Section 3.14); Section 4.1 discusses this alongside other broader methodological risks evident across the reviewed literature. To address this, we recommend a minimum of 100 participants, in line with the participant number typically used in ISO 15197:2013 user-performance evaluations [78], to ensure sufficient diversity. We present this explicitly as a pragmatic convention, not a value derived from a statistical or model-adequacy calculation. Validation estimates obtained from small samples are also biased and imprecise [85]. More samples for training a deep learning model can be created either through equal repeated measurements per person or through proper data-splitting techniques. We recommend that at least 10% of participants in the validation and testing sets have confirmed hyperglycaemia or hypoglycaemia. This is important for a fair evaluation of performance at the glucose extremes where accuracy matters most.

Skin pigmentation, using the Fitzpatrick scale or an objective melanin index, needs to be reported for each participating subject, since melanin content is a known, independent influence on optical absorption and scattering [86,87]. Demographic variables such as age, BMI, and sex should be recorded, but, as discussed above, should not be used as direct inputs to the trained predictive model. This is because models trained on superficial, non-causal correlates are known to fail once those correlates no longer hold in a new population [28]. The reviewed literature also provides direct demonstrations of the risk; Sen Gupta et al. [32] report that BMI alone was more strongly correlated with their training labels than any feature extracted from the pure optical signal. Diabetes status, duration, and relevant coexisting conditions such as vascular disease or neuropathy should also be reported if present. Skin blood flow and perfusion can affect both PPG and non-invasive glucose measurement [88,89]; environmental and skin surface temperature can also affect photoplethysmographic signals [90]. Therefore, heart rate, blood pressure, temperature, motion (for wearable instrumentation), and the exact placement of the measurement site should be reported for a meaningful cross-study comparison.

During the implementation and evaluation of machine-learning approaches, subject-wise validation should be used and explicitly reported as such. We recommend that authors report accuracy separately for the training set, an internal validation set, and an unseen test set, because the difference between these three figures is itself informative. A model whose training accuracy is much higher than its validation or test accuracy is a clear sign of overfitting, and reporting only a single combined figure conceals this. We recommend subject-wise k-fold cross-validation when the dataset contains fewer than 1000 samples. In addition, we recommend prospective evaluation on at least 50 samples from an external cohort that was not used during training or validation, wherever the study design allows it. Section 3.13 shows why this matters: only 2 of the 37 studies reported any real-time or prospective testing, and neither provided sufficient detail to assess external validity.

To improve generalisability, participant recruitment should also include, where possible, patients with conditions that can alter blood physiology. Examples include anaemia, peripheral vascular disease, and renal impairment. Excluding these patients risks overestimating device accuracy in exactly the populations where non-invasive monitoring is needed most clinically. Researchers reusing previously published datasets must ensure, during deployment, that instrumentation specifications, and particularly the sampling frequency of the new device, match those of the original device used for data collection. Glucose-dependent secondary changes in the optical properties of skin are real, but they are not sufficient on their own for reliable glucose measurement. The physical, chemical, and physiological basis of selectivity must be understood. In addition, the standalone contribution of the optical channel to accuracy should be demonstrated with an ablation study whenever a multimodal or sensor-fusion approach is used. The optical path length, signal-to-noise ratio, and the pressure between the sensor and the measurement site should be fixed, reproducible, and explicitly reported in the methods section.

The recommendations are summarised in Table 3. There are two distinct groups. The first group is motivated by gaps observed in the quantitative findings of this review (R). It includes reporting the exact reference device and procedure, stating the validation strategy explicitly and at the subject level, recording demographic variables without using them as model inputs, reporting error-grid analysis (Clarke and Parkes) alongside point-error metrics, and testing on external or prospective data. The second group comprises pragmatic recommendations (P) based on broader methodological considerations, in which the numerical value serves as a working threshold rather than a value derived from this review. This applies to the minimum of 100 participants, the minimum of 10% hypo- or hyperglycaemic participants, the 20-second acquisition time, the 50-sample external test, and the use of k-fold cross-validation below 1000 samples. Our data show that small cohorts (27 of 37 studies under 100 participants) and limited coverage of hypoglycaemia (only 9 of 37 studies with values below 70 mg/dL) are common, but they cannot identify the number at which a study becomes adequate. We recommend 100 participants and the inclusion of 10% hypo/hyperglycaemic cases because, in this way, the diversity of the dataset can be ensured with regard to inter-subject variability (anatomical, anthropometric, physiological), and publicly available datasets can then be integrated for designing and evaluating more data-hungry models. Commercial devices named in the framework, such as the Accu-Chek Guide (capillary meter) and the FreeStyle Libre 3 (a continuous glucose monitor, which measures interstitial glucose and serves as a secondary comparator), are examples of suitable options and not universally preferred gold standards. We propose these to allow studies to be compared on equal scale and to enable future work to test and revise them, rather than as a scientific claim for achieving guaranteed robust performance.

This framework is not intended to serve as a clinical-trial protocol or as a regulatory approval requirement. Sample-size and validation standards for regulatory clearance of a medical device are set by regulatory bodies such as the FDA or under ISO/IEC standards, and are outside the scope of this review.

### 4.4. Novelty and Importance of This Study

Villena Gonzales, Mobashsher, and Abbosh [5] reviewed the full range of glucose monitoring technologies, including invasive, minimally invasive, and non-invasive methods, with strong breadth but without a systematic search protocol and without depth on optical or ML-based approaches specifically, which now dominate this literature.

Jernelv et al. [92] explained the light-tissue interaction underlying near-infrared, mid-infrared, Raman, photoacoustic, polarimetric, fluorescence, and optical coherence tomography sensing, but predates the widespread use of deep learning and wearable ML-based systems in this field and does not follow a systematic search protocol.

Hina and Saadeh [93] reviewed near-infrared technology for glucose monitoring, with a particular focus on NIR photoplethysmography and covered wavelength choices, feature extraction, and ML approaches. However, the review is limited to near-infrared sensing, does not compare it against other optical modalities, and proposes no common framework.

Xi and Gong [94] reviewed five optical techniques applied specifically to the aqueous humour of the eye, addressing sensing physics, instrumentation, and feasibility at that single anatomical site, but not at the skin, earlobe, or fingertip sites used by the majority of studies in our corpus, and without addressing machine-learning implementation.

Most recently, Jiang, Yao, and Ding [29] reviewed PPG-based glucose sensors specifically. This review is closely aligned with the PPG-specific subset of our own corpus, but, by design, does not extend to NIR, Raman, photoacoustic, OCT, or polarimetry studies, and does not classify each study for shortcut-learning risk, which we do here using an author-defined screening rubric.

Chan et al. [27] performed a scoping review of AI-based glucose monitoring and applied a formal, published quality-assessment instrument for medical AI research to 33 included studies. They reported an average quality score of only 33.5 out of 100. Chan et al. [27] considered studies published until 8 February 2023, while we considered studies published until August 2026. Chan et al. [27] scored each study against a fixed medical-AI checklist and produced a single quality score per study. By design, it does not name the specific mechanism by which a given study accuracy might be inflated, and it is not restricted to optical sensing modalities. For each of the 37 optical studies in our review, we identify which specific factor drives its risk: small sample size, a demographic variable used as a direct model input, an unconfirmed subject-wise split, or an absent real-time test. We also indicate which items of our proposed framework are motivated by gaps documented in this evidence base and which are pragmatic working thresholds. Where Chan et al. tell readers that AI-based glucose monitoring research has a quality problem in general, our review is intended to tell readers the cause behind this issue, how the problem can be solved, and how scientific contribution can be established.

### 4.5. Limitations of This Review

This review has several limitations that should be considered when interpreting its findings. The quality-score threshold for inclusion (63/100) was chosen after the scores had been examined, not before, so it may introduce selection bias, and our conclusions describe the higher-scoring part of the literature rather than the literature as a whole. To check how far the conclusions depend on this value, we repeated the main summaries with a stricter cut-off of 68 (26 studies) and a looser cut-off of 58 (40 studies). The main conclusions were unchanged in both analyses.

At a cut-off of 68 (26 studies), hold-out remained the most common validation method (11 studies, versus 8 using k-fold cross-validation), none of the studies reported real-time testing, and at least 65% still enrolled fewer than 100 participants. A high risk of shortcut learning was assigned to 62% of the studies. The reference-measurement problem also persisted: 13 studies used a commercial invasive glucometer alone as the reference, 7 used laboratory or venous analysis, 4 used a continuous glucose monitor, and 2 reported no reference device. Only 8 of 26 studies (31%) included readings below 70 mg/dL, and 5 of 26 (19%) used demographic or anthropometric variables as model inputs.

Similarly, at a cut-off of 58 (40 studies), hold-out remained the most common validation method (21 studies, versus 11 using k-fold cross-validation). Only 3 of 40 (8%) reported any real-time or deployment testing, and at least 65% still enrolled fewer than 100 participants. A high risk of shortcut learning was assigned to 63% of the studies (25 of 40). The reference-measurement problem also persisted: 21 studies used a commercial glucometer alone as the reference, 10 used laboratory or venous analysis, 5 used a continuous glucose monitor, and 5 reported no reference device. Only 9 of 40 studies (23%) included confirmed readings below 70 mg/dL, and 9 of 40 (23%) used demographic variables as model inputs. The main conclusions were therefore not sensitive to the exact cut-off.

In our current review, several sensing modalities, notably OCT, polarimetry, and hybrid mmWave approaches, are represented by only one or two studies each. Conclusions drawn about these modalities specifically should therefore be treated as provisional. Our shortcut-learning risk classification was applied by the review authors based on each study’s reported methodology. It is an author-defined screening rubric rather than a validated, externally standardised instrument, and the resulting ratings are judgements made by the review authors, and a different research team applying the same rubric could reach different judgments on borderline cases. The shortcut-learning ratings were assigned by one reviewer and verified against the source papers by a second, rather than being rated independently by two reviewers, so inter-rater agreement could not be calculated. The proposed framework is a set of recommendations synthesised from this evidence base and from general machine-learning and biomedical-engineering practices. It has not been prospectively tested. We do not claim that a study following it would necessarily achieve better real-world accuracy. We only claim that it would allow a reader to assess more directly whether reported accuracy reflects a robust performance or not. Although our search was conducted across six databases with documented Boolean strings, it may still have missed relevant studies. These could include studies published in venues outside these databases, in languages other than English, or as conference proceedings not indexed by these sources.

## 5. Conclusions

We conducted a systematic review of AI-enabled optical sensing devices for non-invasive blood glucose monitoring. Based on the defined search and quality assessment criteria, studies focusing on photoplethysmography, NIR, mid-IR, Raman spectroscopy, photoacoustics, optical coherence tomography, and hybrid approaches were included in this review. Using an author-defined three-tier screening rubric, whose ratings reflect the review authors’ own judgements rather than a validated risk-of-bias assessment, we rated 62% of the studies as High risk of shortcut learning. Under our rubric, the most frequent driver of a High rating was the direct use of demographic variables such as BMI or age as model inputs, often together with near-perfect accuracy reported from very small cohorts. Beyond the rubric itself, the reviewed literature also showed low specificity and sensitivity for glucose at the most commonly used wavelengths and a near-complete absence of real-time testing, both of which raise further concern about the generalisability of reported accuracy across the corpus as a whole. In contrast, Raman spectroscopy showed the lowest shortcut-learning risk (four of the five Raman studies were rated Low), which is consistent with a more direct physical link to glucose absorption (with only five studies, the finding is provisional). Based on our findings, we developed a common framework that proposes recommended criteria for signal acquisition, subject recruitment/data collection, and performance reporting. This helps ensure data reusability and allows NIBGM research groups to compare results and collaborate more meaningfully across future studies.

## Figures and Tables

**Figure 1:**
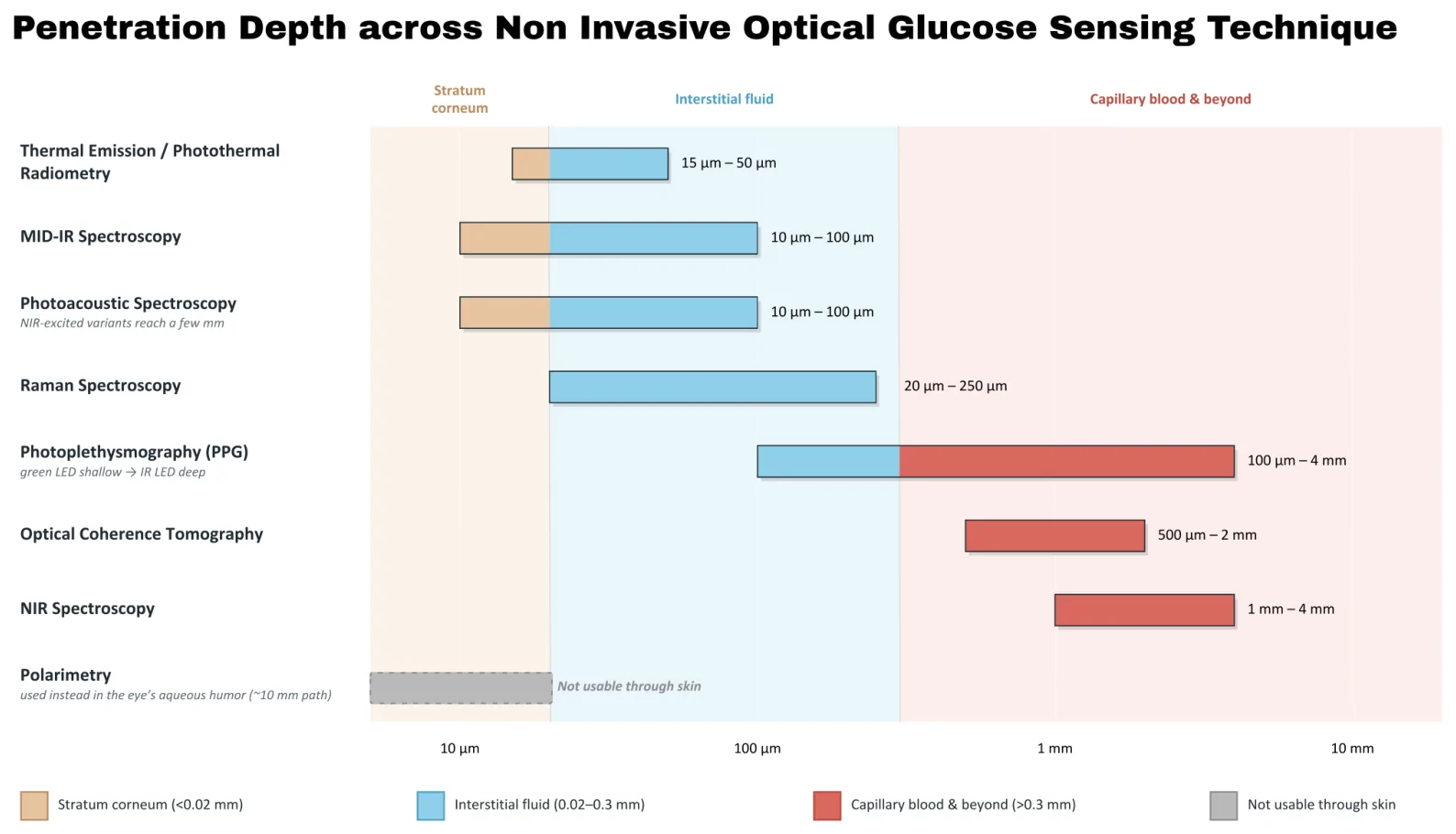
Penetration depth across the non-invasive glucose sensing technique.

**Figure 2:**
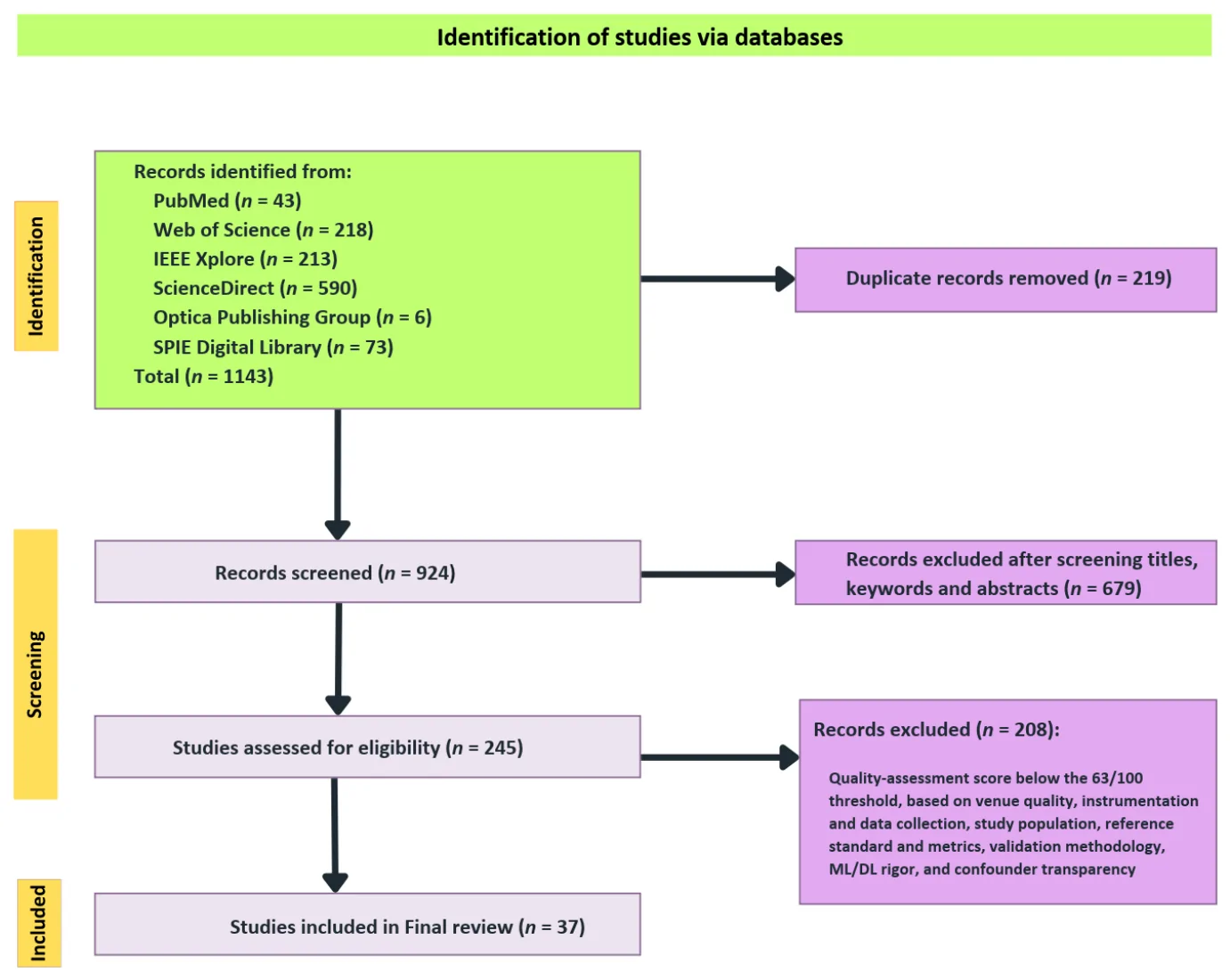
PRISMA 2020 flow diagram showing the identification, screening, eligibility, and inclusion of studies.

**Figure 3:**
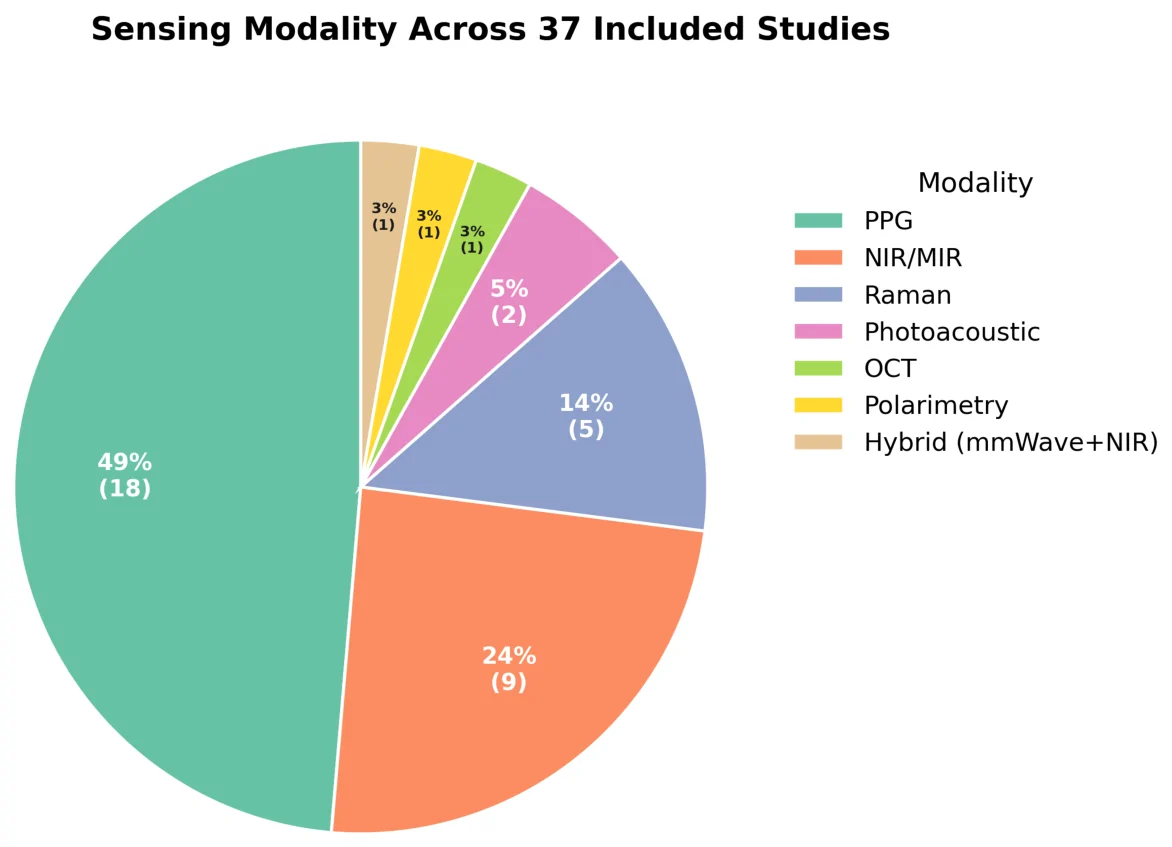
Sensing modality across the 37 included studies. Counts: PPG 18 (49%); NIR/MIR 9 (24%); Raman 5 (14%); photoacoustic 2 (5%); OCT 1 (3%); polarimetry 1 (3%); hybrid mmWave+NIR 1 (3%). Percentages are rounded individually to the nearest whole number.

**Figure 4:**
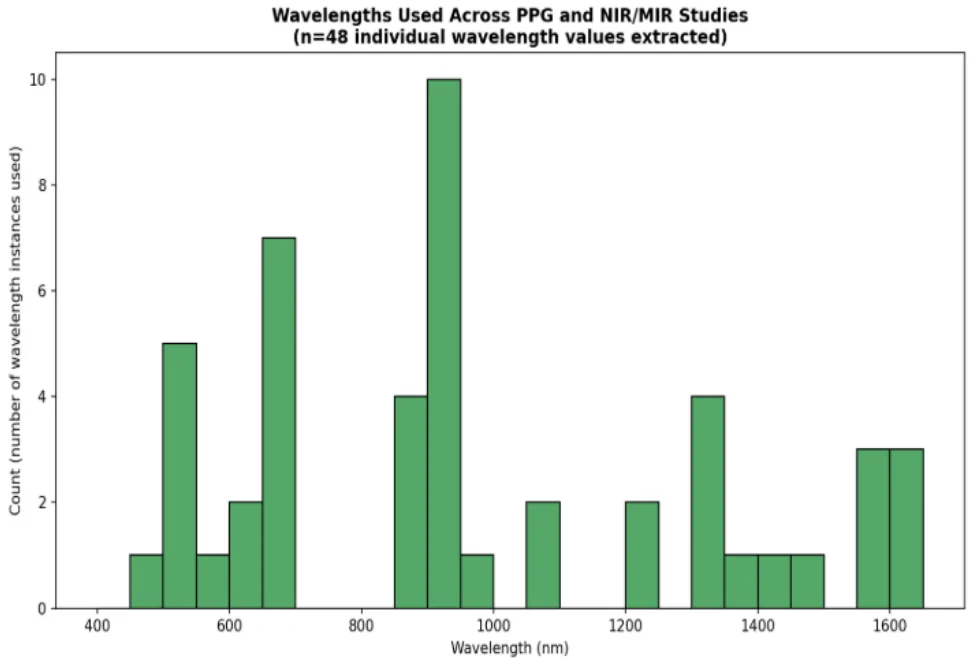
Distribution of individual wavelength values reported across the 27 PPG and NIR/MIR studies (48 individual wavelength values extracted, since many studies report more than one).

**Figure 5:**
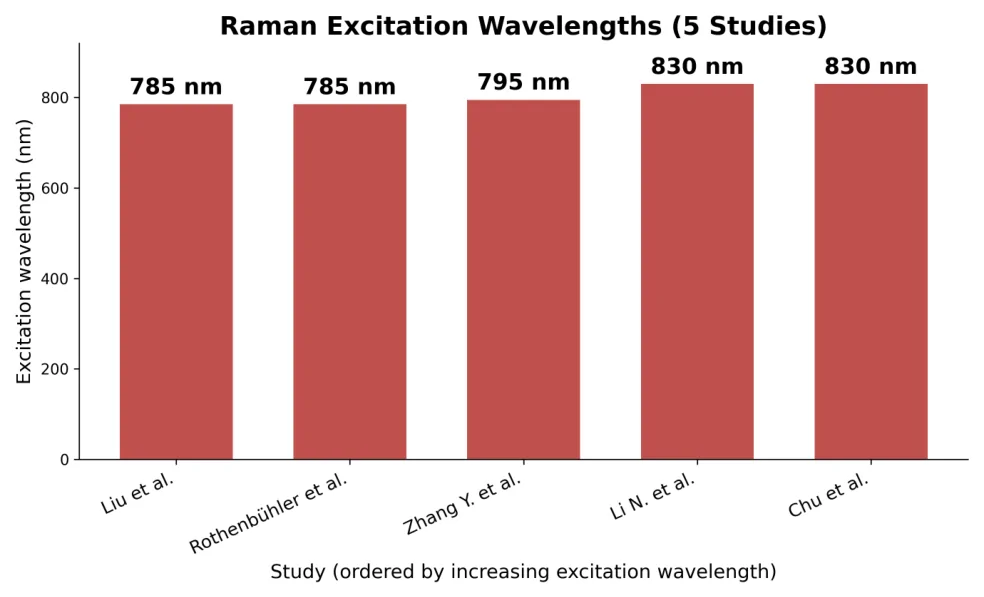
Excitation wavelength used in each of the 5 Raman spectroscopy studies (Li et al., 2019 [59]; Liu et al., 2025 [60]; Zhang et al., 2025 [61]; Rothenbühler et al., 2026 [62]; Chu et al., 2026 [63]).

**Figure 6:**
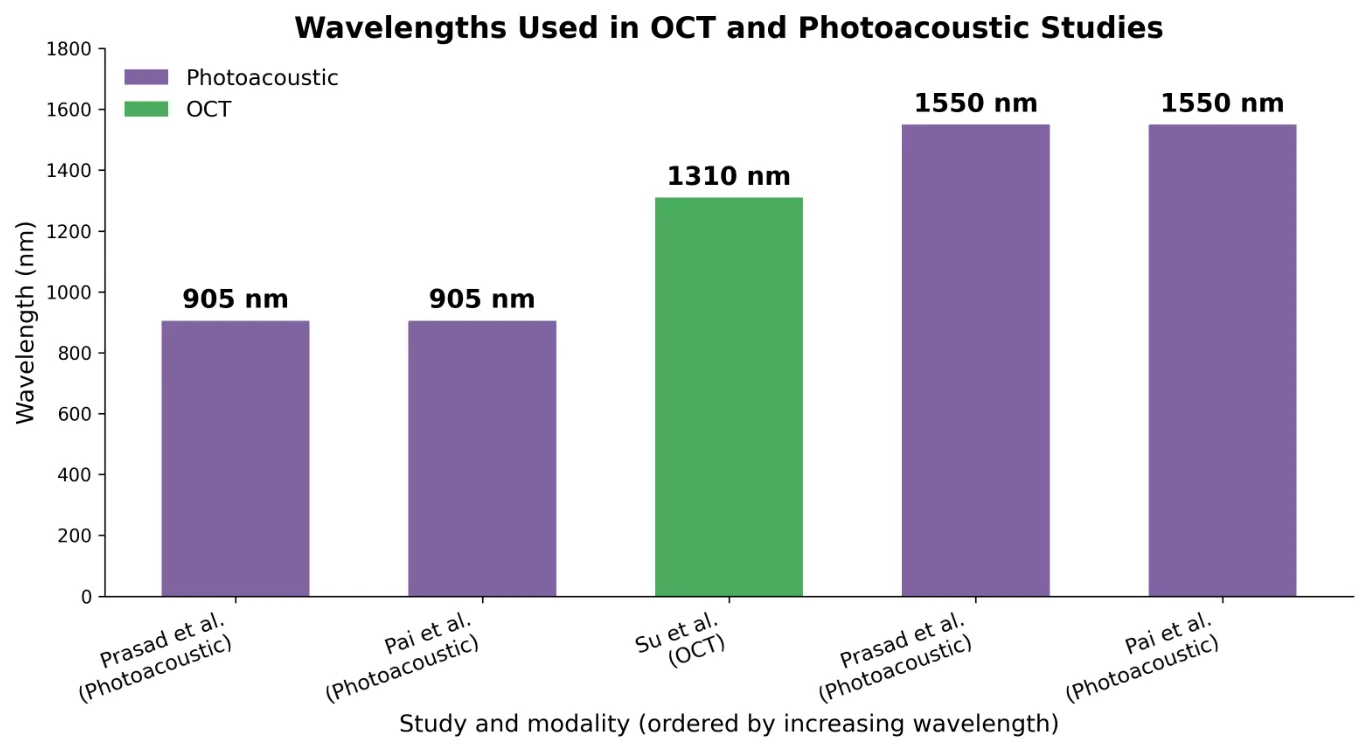
Wavelength(s) used in the 2 photoacoustic studies (Prasad et al., 2025 [64]; Pai et al., 2018 [65]) and the 1 OCT study (Su et al., 2025 [66]).

**Figure 7:**
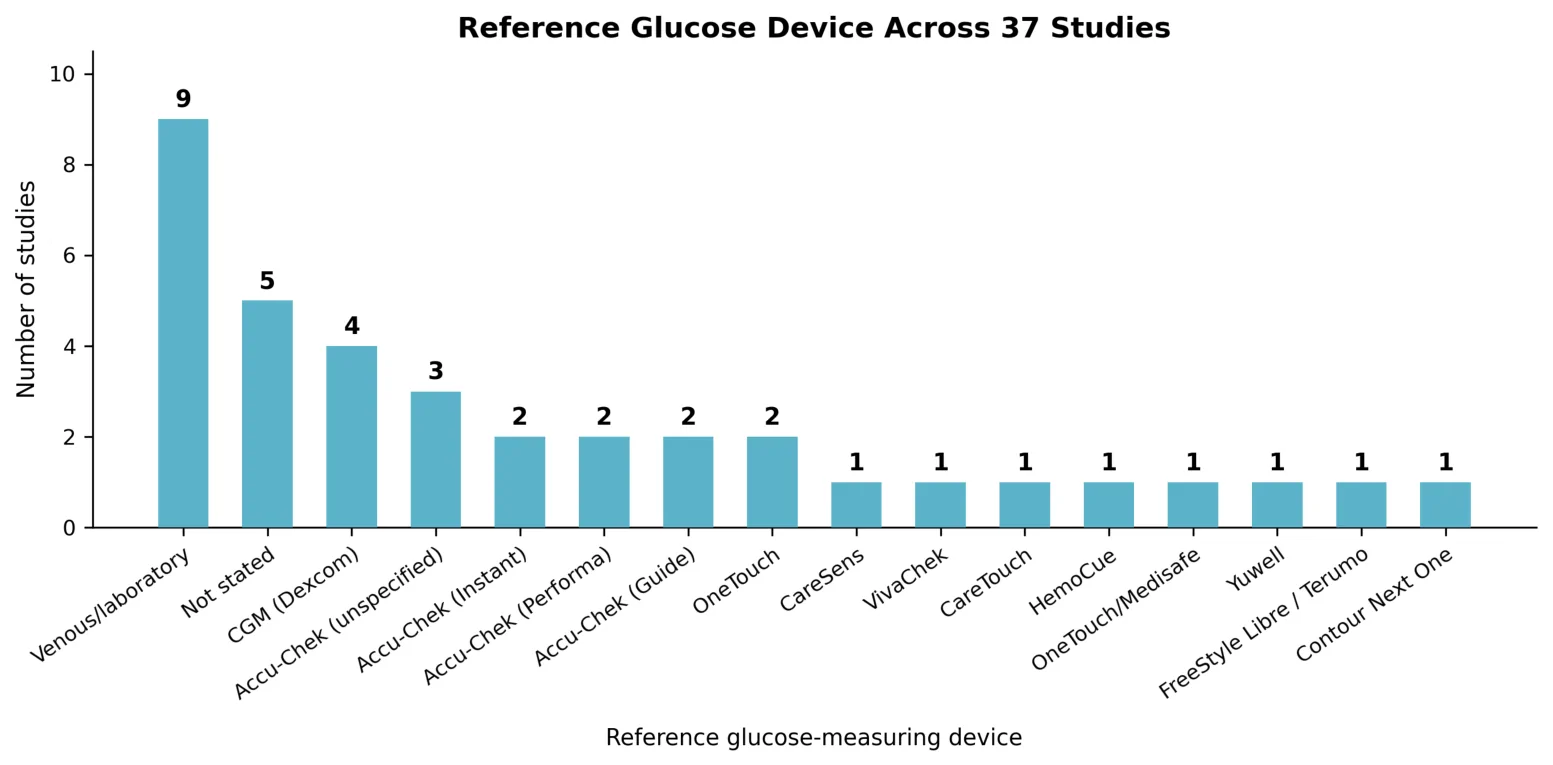
Reference glucose-measuring device used to validate each of the 37 studies.

**Figure 8:**
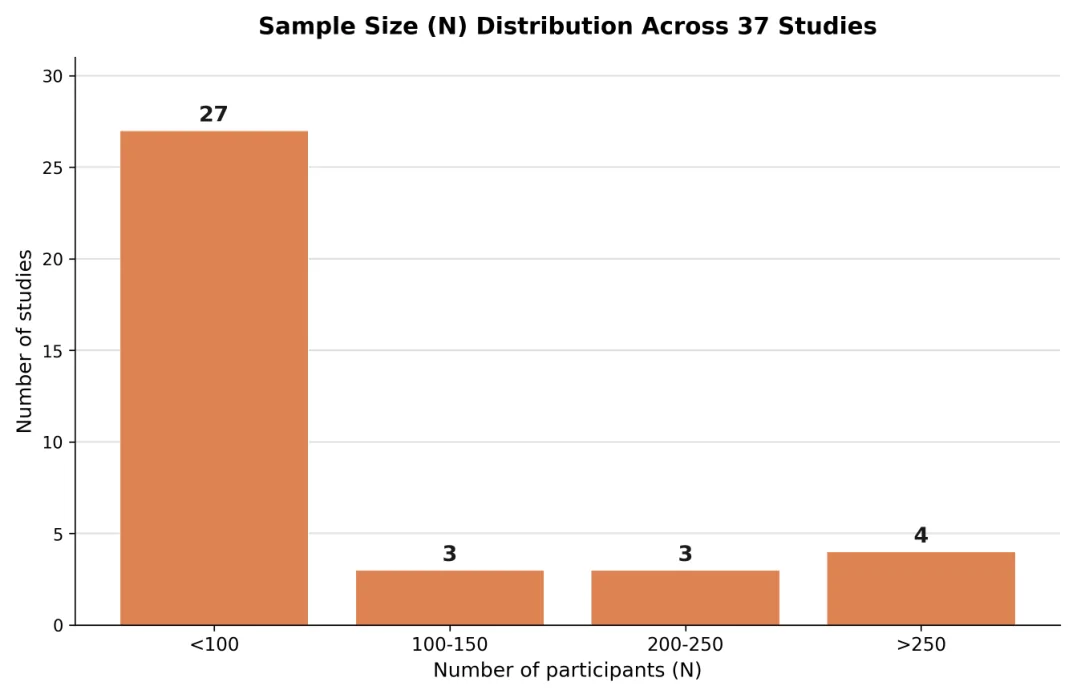
Number of participants (N) across the 37 included studies.

**Figure 9:**
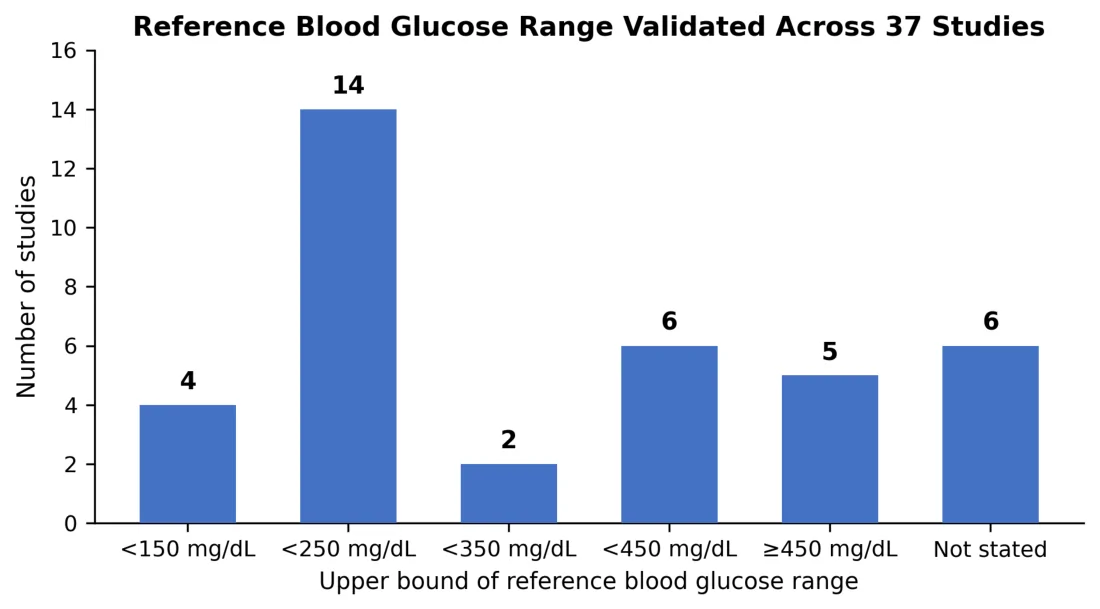
Span of the reference blood glucose range validated in each study.

**Figure 10:**
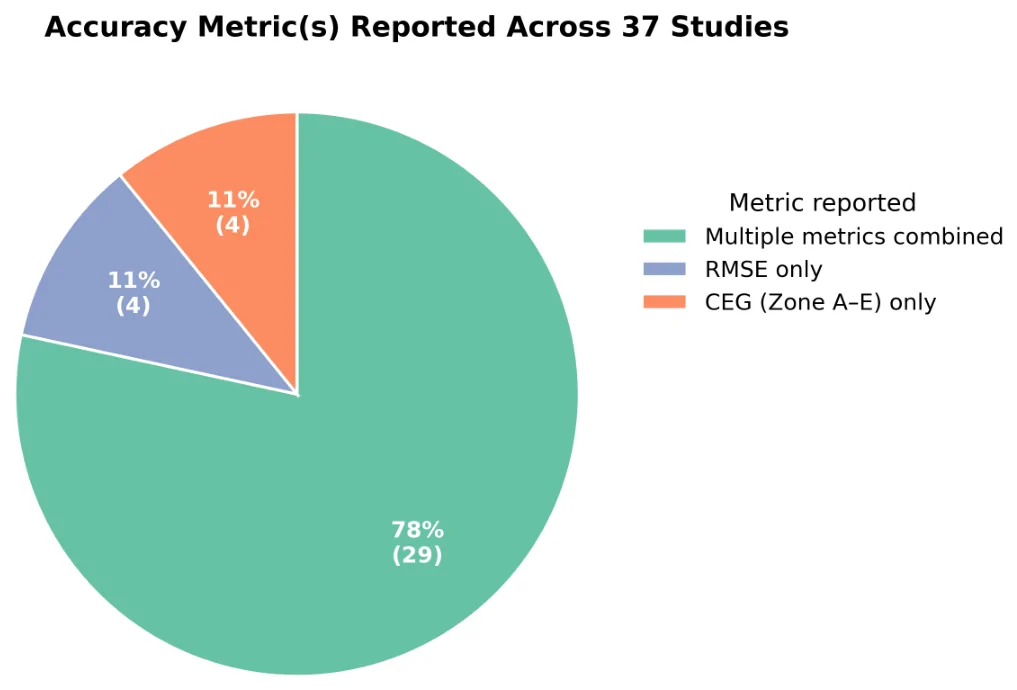
Accuracy metrics reported by each study.

**Figure 11:**
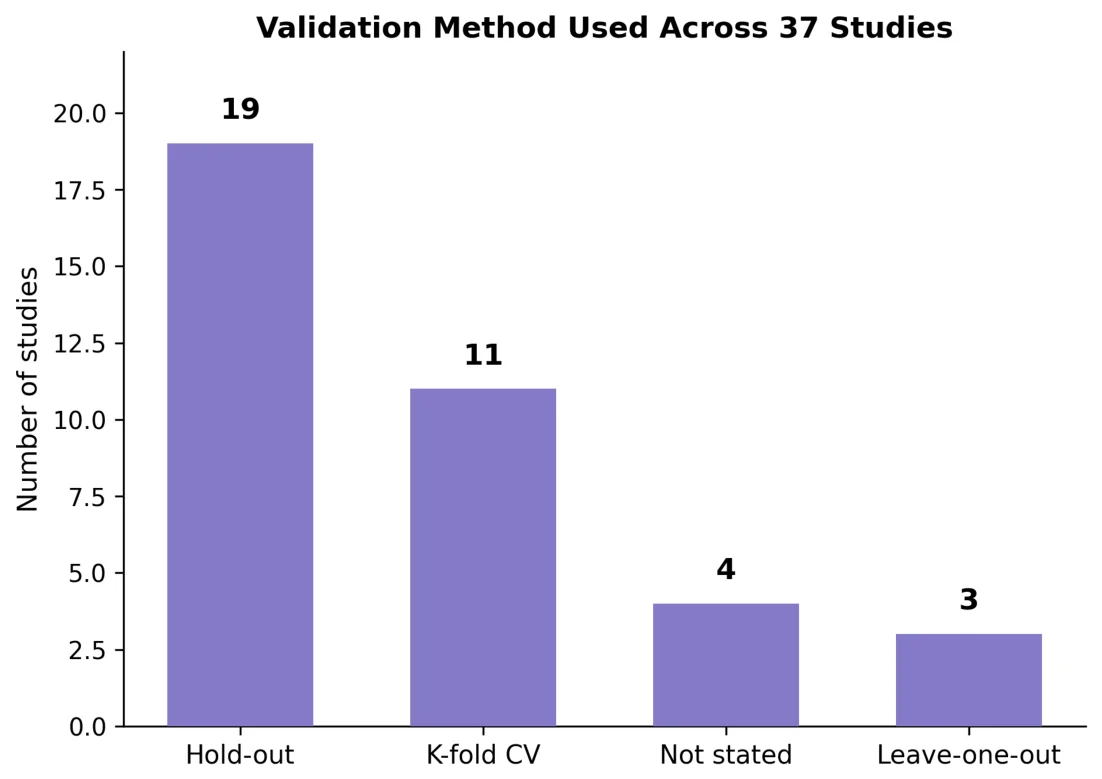
Validation method used across the 37 included studies.

**Figure 12:**
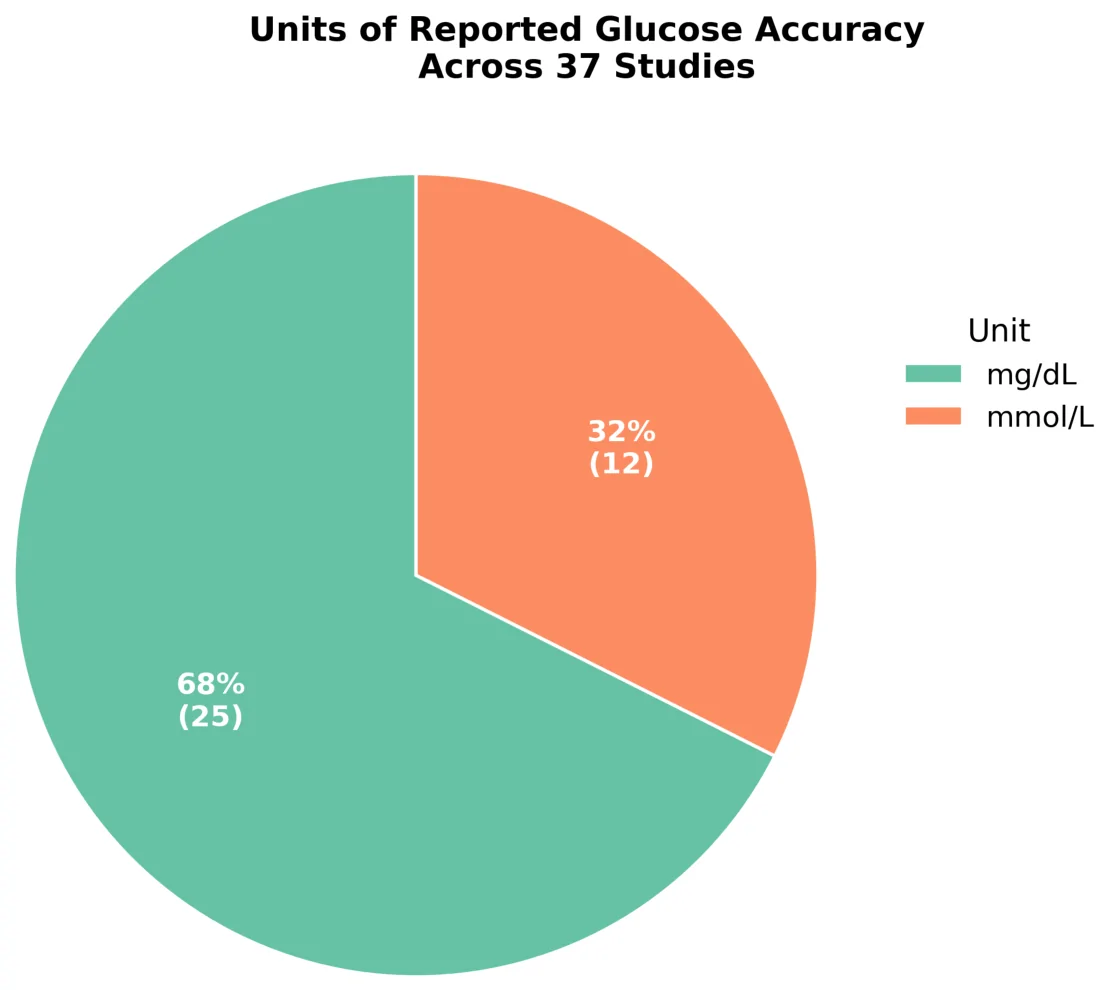
Units used to report glucose prediction accuracy.

**Figure 13:**
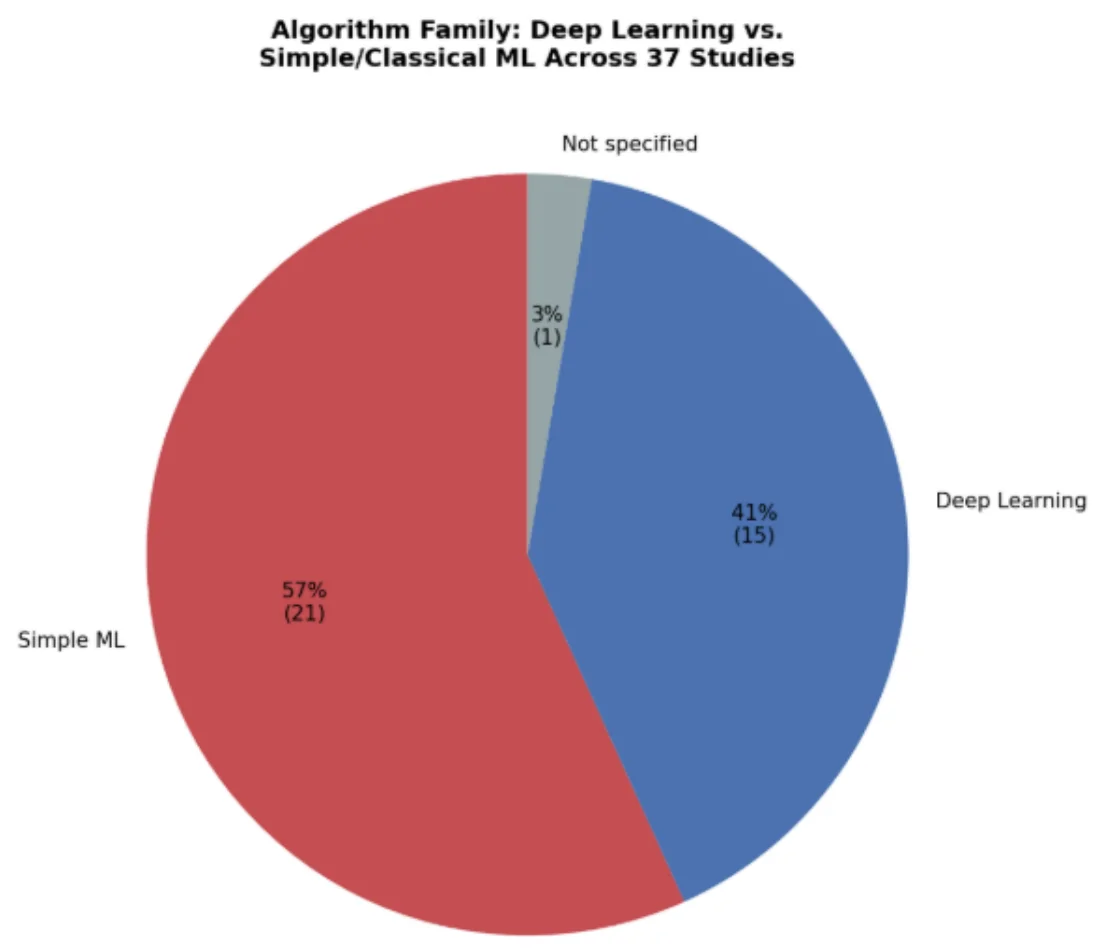
Algorithm family used across the 37 included studies. Counts: Simple/classical ML 21 (57%); deep learning 15 (41%); not specified 1 (3%). Percentages are rounded individually to the nearest whole number.

**Figure 14:**
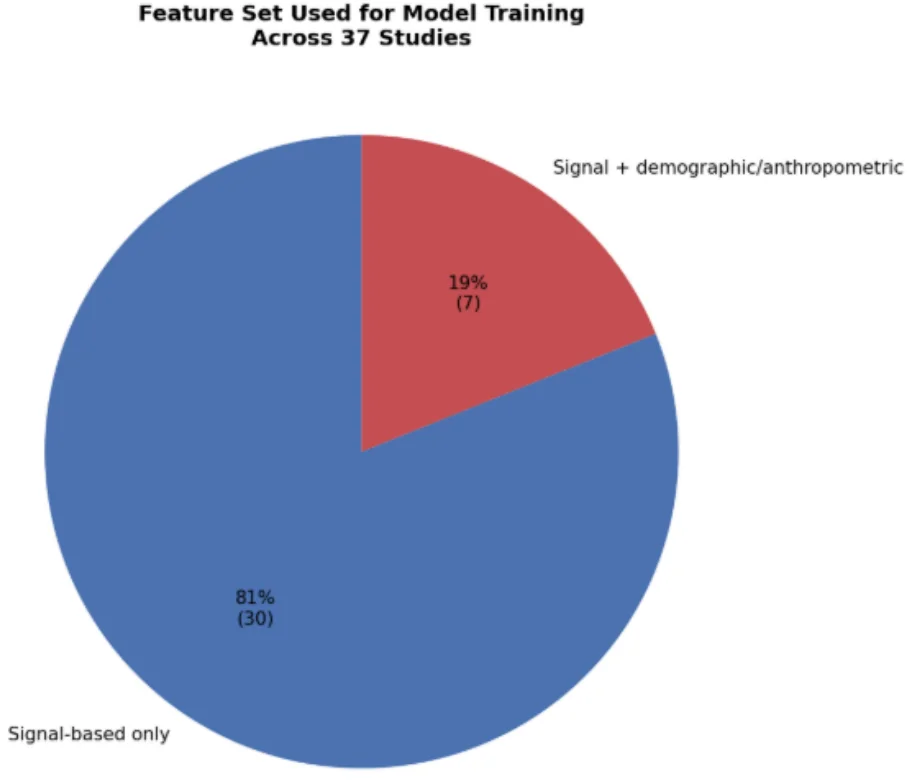
Whether each study’s trained model used signal-derived features only, or signal features plus an explicit demographic/anthropometric covariate.

**Figure 15:**
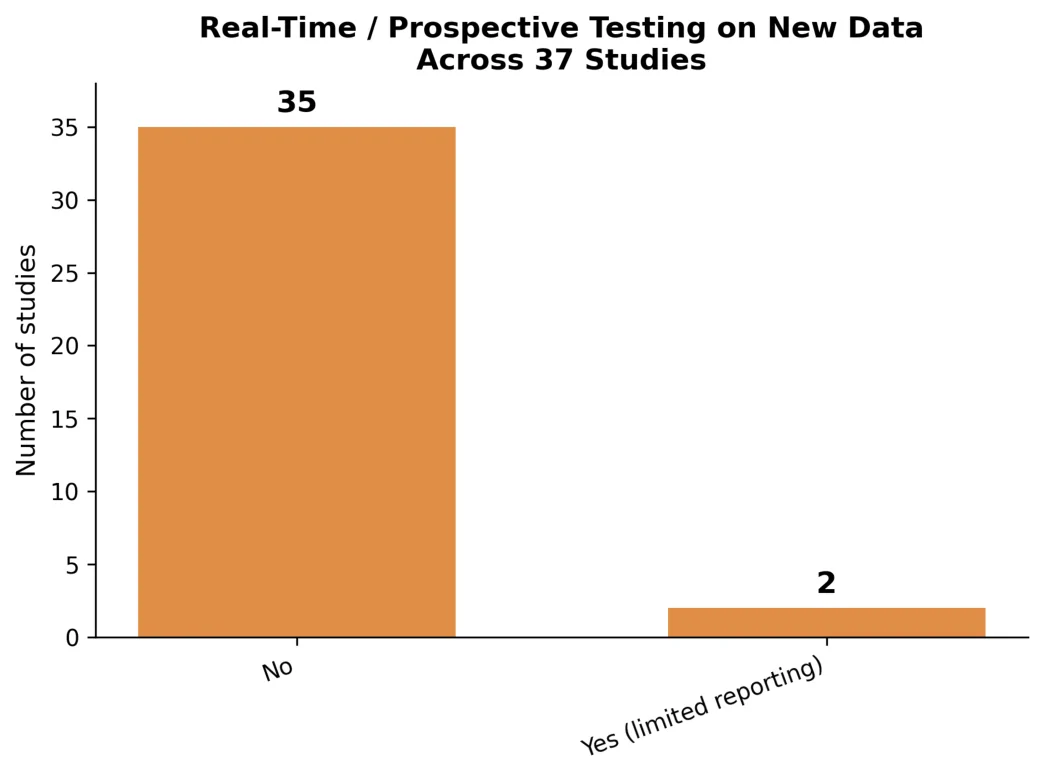
Whether each study performed real-time or prospective testing beyond its offline train/test evaluation.

**Figure 16:**
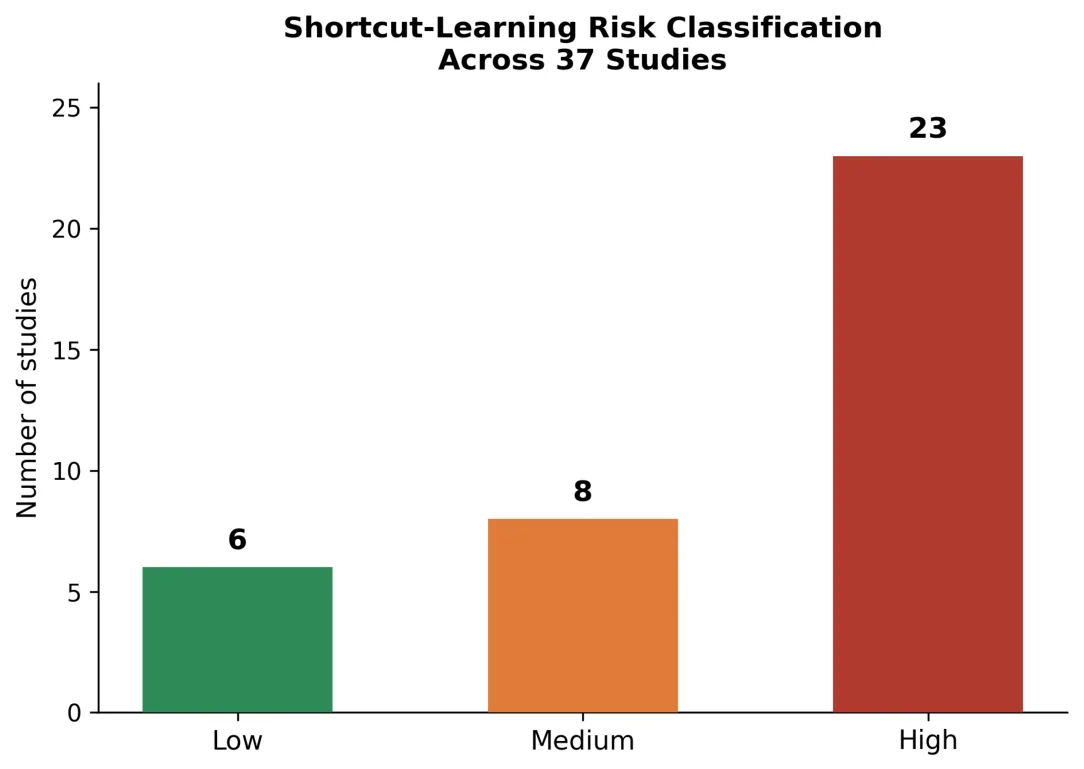
Shortcut-learning risk classification across the 37 included studies (Low, Medium, and High risk). The classification uses an author-defined screening rubric (Section 3.14); it is not a validated risk-of-bias instrument, and the ratings shown are the review authors’ own judgements.

**Table 1:** Different non-invasive techniques for blood glucose monitoring.

Measuring Technique	Working	Advantages	Disadvantages
**NIR Spectroscopy**	This approach is based on the absorption as well as the scattering of wavelengths in the range of 780 nm to 2500 nm, which is caused by internal bond rotation and molecular vibrations [11].	Measurement is direct on the skin. No need for sample preparation. Supports real-time monitoring. Miniaturised and simple hardware. Can support a multiwavelength approach [12].	Glucose’s NIR absorption bands are weak and broad. Skin temperature affects the scattering and absorption properties. Susceptible to motion artefacts Interference with skin pigmentation. Sensor drift because of blood perfusion and hydration.
**MID-IR Spectroscopy**	It uses light in roughly the 2500–25,000 nm range. It measures fundamental vibrational absorption bands of molecules, not the weaker overtone bands used in NIR [13].	Stronger glucose absorption in MIR than in NIR. Less scattering, cleaner signal. More chemical specificity than NIR.	High absorption by water molecule. Cannot penetrate deeper into the tissues. (Penetration depth: 10 µm using DRIFTS, 2.5–25 µm range.) Results vary due to skin thickness. Expensive Instrumentation.
**Raman Spectroscopy**	Raman spectroscopy measures inelastic scattering. By shining a laser on the skin, scattered light is detected to measure a shifted wavelength (the Raman shift). This shift is caused by molecular vibrations, and each molecule has its own distinct pattern [14].	Raman gives a highly specific fingerprint for glucose. No reagents or consumables are needed. Repeated measurements on the same spot are possible. Can reach interstitial glucose easily.	Signals are intrinsically very weak. Skin fluorescence can blurry glucose signal. Long laser exposure can risk safety and comfort. Results can be shifted with skin thickness, hydration, and probe pressure, etc.
**Optical Coherence Tomography**	OCT measures elastic backscattering versus depth. OCT sends low-coherence light into the skin and uses an interferometer to detect the light that scatters back from different depths. As glucose concentration rises, it changes the refractive index of interstitial fluid. This changes the tissue’s scattering coefficient, which shows up as a change in the OCT signal slope [15].	Specific skin layer can be targeted through OCT. One measurement can separate signals from different tissue layers. Detection sensitivity for backscattered signals is very high.	Scattering coefficient changes from glucose are small and can be masked by hydration or other interferents. It does not capture direct glucose signature. OCT systems are relatively bulky and expensive.
**Photoplethysmography (PPG)**	Measures the variation in light transmitted through or reflected from tissue as arterial blood volume changes with each cardiac cycle; glucose-related information is extracted from subtle changes in the pulsatile (AC) and non-pulsatile (DC) components by using one or more LEDs in the visible-to-near-infrared range [16].	PPG hardware is very cheap and has miniaturisation potential. It consumes very little power and can be easily integrated into wearable systems. It provides real-time signal acquisition and a high sampling rate.	The glucose-specific part of the PPG signal is very weak. Motion artefacts and skin temperature affect the signals. Skin pigmentation and perfusion change the DC level of the PPG signal.
**Polarimetry**	When linearly polarised light passes through a glucose containing medium, glucose rotates the plane of polarisation. The angle of this rotation is directly proportional to both the glucose concentration and the length of the optical path through the medium [17].	Direct, well-understood physical relationship between rotation angle and glucose concentration. Performs well in optically clear media such as the aqueous humour of the eye.	Skin and other highly scattering tissues depolarise light strongly and are unworkable targets for it [18]. Has less selectivity due to other interfering agents Glucose signal is very low and need highly sensitive instrumentation.
**Photoacoustic Spectroscopy**	A pulsed or modulated laser (usually mid-infrared) is absorbed by tissue. This absorbed light causes tiny, rapid heating, which makes the tissue expand and contract. That expansion creates an ultrasonic pressure wave, which a transducer detects. The strength of the acoustic signal is directly related to how much light was absorbed [19].	PAS reaches deeper than plain mid-infrared spectroscopy because the acoustic wave carries the signal out through tissue instead of relying on light to travel back out. Glucose has strong, specific absorption peaks in the mid-infrared, from C-H-O bond vibrations. Depth-gating optoacoustic signals have lower interference with superficial skin tissues [20].	Instrumentation is complex and bulky. The practical mid-infrared PAS penetration is generally under 100 µm, so it still only reaches the outer interstitial fluid layer, not deeper capillaries Skin conditions can cause signal variability.
**Thermal Emission/Photothermal Radiometry**	Two mid-infrared laser beams shine on the skin at the same time; one tuned to the peak of glucose absorption band (9600 nm), the other tuned to a nearby baseline wavelength (10,500 nm) where glucose does not absorb as strongly. The two beams are modulated 180° out of phase with each other. This absorbed light heats the tissue by a tiny amount, and the tissue re-emits that heat as modulated infrared radiation [21].	As the two beams were out of phase, the interference effect could be reduced significantly. Sensitivity can be tuned by adjusting the intensity ratio and phase difference, and thus variability among different skin types can be reduced.	As it uses mid-infrared excitation, penetration depth is shallow. Due to the need for precise and stable control of two lasers, delicate instrumentation is required. Validated results are during in-vitro conditions [21].
**Microwave Sensing**	One antenna system emits radiation with a frequency in the gigahertz range while another system receives it and detects the changes in its electrical characteristics caused by blood glucose level [22].	Microwaves can penetrate deeper into tissues. (At 2.45 GHz, depth is 17 mm; can reach the dermis, fat, and upper muscle layer.) Small glucose changes can be detected. Does not cause physiological ionisation.	This method is also sensitive to fluctuating physiological conditions, such as cardiorespiratory functions, which can lead to inaccuracy. Dielectric changes are not unique to glucose [23].
**Bioimpedance Spectroscopy (BS)**	Blood glucose levels can affect the permittivity of red blood cells; thus, the capacitance will be changed accordingly. Changes in the impedance can be determined by applying a known amount of AC [24].	It is comparatively a much simpler and cost-effective technique. Information from several different tissue compartments in a single measurement can be extracted by changing frequencies.	It could be affected by fluctuations in physiological conditions and is also sensitive to sweat, temperature, and motion. Dry electrodes’ contact quality is affected by skin roughness, hydration state, and contact pressure, which introduces measurement variability between sessions.
**Ultrasound**	It works on the time-of-flight method. The velocity is directly related to glucose concentration, and TOF decreases because of higher glucose levels [25].	It penetrates deeper into the tissues and thus can be used for extracting blood glucose information. External features of a body, such as skin colour, are not hindrances to its performance.	Temperature can affect the speed of sound. This method is not sensitive to only blood glucose levels.

**Table 2:** Full Boolean search string used for each database.

Database	Full Search String
PubMed	(("non-invasive"[tiab] OR "noninvasive"[tiab]) AND ("blood glucose"[tiab] OR "glucose monitoring"[tiab] OR "glucose sensing"[tiab])) AND ("optical"[tiab] OR "NIR"[tiab] OR "near-infrared"[tiab] OR "mid-infrared"[tiab] OR "PPG"[tiab] OR "photoplethysmography"[tiab] OR "Raman"[tiab] OR "OCT"[tiab] OR "optical coherence tomography"[tiab] OR "optoacoustic"[tiab] OR "photoacoustic"[tiab] OR "polarimetry"[tiab]) AND ("artificial intelligence"[tiab] OR "machine learning"[tiab] OR "deep learning"[tiab] OR "neural network"[tiab] OR "signal processing"[tiab] OR "algorithm"[tiab] OR "regression"[tiab]) AND ("experimental"[tiab] OR "pre-clinical"[tiab] OR "clinical"[tiab] OR "in-vivo"[tiab] OR "in-vitro"[tiab])
Web of Science	TS = (("non-invasive" OR "noninvasive") AND ("blood glucose" OR "glucose monitoring" OR "glucose sensing")) AND TS = ("optical" OR "NIR" OR "near-infrared" OR "mid-infrared" OR "PPG" OR "photoplethysmography" OR "Raman" OR "OCT" OR "optoacoustic" OR "photoacoustic" OR "polarimetry") AND TS = ("artificial intelligence" OR "machine learning" OR "deep learning" OR "neural network" OR "signal processing" OR "algorithm" OR "regression") AND TS = ("experimental" OR "pre-clinical" OR "clinical" OR "in-vivo" OR "in-vitro")
IEEE Xplore	("non-invasive" OR "noninvasive") AND ("blood glucose" OR "glucose monitoring") AND ("optical" OR "NIR" OR "PPG" OR "photoplethysmography" OR "Raman" OR "OCT" OR "photoacoustic" OR "polarimetry") AND ("machine learning" OR "deep learning" OR "artificial intelligence" OR "neural network" OR "algorithm")
ScienceDirect	("non-invasive" OR "noninvasive") AND ("blood glucose" OR "glucose monitoring") AND ("optical" OR "NIR" OR "PPG" OR "Raman" OR "OCT" OR "photoacoustic") AND ("machine learning" OR "deep learning" OR "artificial intelligence")
SPIE Digital Library	(Blood Glucose) AND (Non-Invasive) AND (Machine Learning), the exact-phrase Boolean string used for other databases returned zero results on this platform.
Optica Publishing Group	("non-invasive" OR "noninvasive") AND ("blood glucose" OR "glucose monitoring" OR "glucose sensing") AND ("optical" OR "NIR" OR "near-infrared" OR "PPG" OR "photoplethysmography" OR "Raman" OR "OCT" OR "photoacoustic") AND ("machine learning" OR "deep learning" OR "artificial intelligence" OR "neural network")

**Table 3:** Summary of the proposed common framework. Each item is a recommendation for designing and reporting studies that evaluate machine-learning model performance in NIBGM research. Basis: R = directly derived from the quantitative findings of this review; P = pragmatic recommendation, in which any numerical value is a working threshold rather than a value derived from this review.

Item	Proposition	Rationale	Basis
Signal acquisition time	≥20 s	To capture information below 0.5 Hz.	P
Sampling (in case of PPG)	>28 Hz	Maximum frequency of PPG signals currently used is 14 Hz; per the Nyquist theorem, the sampling frequency should be more than double this [80].	P
Reference measurement (capillary)	Use an invasive glucometer approved by relevant regulatory bodies (the Accu-Chek Guide is given as one example; equivalent devices are acceptable)	Distributed in >100 countries [81]; independently reported to exceed ISO 15197:2013 [82]. Section 3.5 shows 37 reviewed studies used at least 12 different reference devices.	R
Reference measurement (continuous)	Where continuous reference is needed (the FreeStyle Libre 3 is given as one example; equivalent devices are acceptable)	Available in >40 countries. Manufacturer-reported MARD of 9.2% in adults [83,84].	R
Number of participants	≥100	A pragmatic convention, not derived from model-adequacy or statistical calculation. Proposed as a simple starting point given the current absence of any common floor in the field for diverse dataset creation.	P
Percentage of hyper-/hypoglycaemic patients during validation and testing	≥10%	To capture extreme BGL values and support a more informative assessment of shortcut-learning risk.	P
Comorbid conditions	Include patients with anaemia, vascular disease, or renal impairment where feasible	To reduce the risk of overestimating device accuracy in the populations where it is most clinically needed.	P
Demographic information	Record BMI, age, and sex for confounder/fairness analysis, but exclude them from the trained model’s feature set	To avoid mistaking demographic correlation for optical sensitivity.	R
Additional participant information	Skin pigmentation (Fitzpatrick scale or objective melanin index)	Melanin content independently affects optical absorption and scattering; recording it helps assess its impact on ML performance.	P
Health status	Diabetes status, vascular disease, neuropathy	These conditions can independently affect the absorption and scattering properties of tissue.	P
Contextual information	Ambient temperature, blood pressure, heart rate, motion artefact, exact measurement-site placement	To account for known confounding influences on the measurement.	P
Validation strategy	Subject-wise validation, explicitly reported as such; report training, validation, and test accuracy separately	Subject-wise validation avoids the inflated performance estimates documented when record-wise splitting is used on data with repeated measures per participant [76]. Reporting training, validation, and test accuracy allows overfitting to be detected directly.	R
Real-time/prospective testing	Evaluate the finalised model on ≥50 samples of an external dataset never used in training or validation, and extreme case data points should also be used where feasible	Real-time testing indicates model robustness; the recommendation follows from Section 3.13 (only 2 of 37 studies did so), while the 50-sample threshold is a working value.	R, P
Accuracy reporting	Report MAE, RMSE, MARD, Parkes Error Grid and a full Clarke Error Grid breakdown, in a stated unit (mg/dL or mmol/L)	Will help in direct comparison across different studies.	R
Ethical approval	Obtain authorisation from an ethics committee; in most cases, a clinical trial authorisation is required	A blood sample, even a capillary, is an invasive procedure under health law, e.g., the French Jarde Act, Article L.1121-1, and required ethical approval [91].	P

## Data Availability

The original contributions presented in this study are included in the article. Further inquiries can be directed to the corresponding author.

## Supplementary Materials

The following supporting information can be downloaded at: https://www.mdpi.com/article/10.3390/s26206572/s1, Supplementary Table S1: the seven-domain, 100-point quality-assessment scoring rubric used for study inclusion (Section 2.5); Supplementary Table S2: study-level detail for all 37 included studies (modality, sample size, reference device, validation strategy, shortcut-learning risk rating, and the specific rubric criterion determining that rating, Section 3.14).
