# Supplementary material for: Non-Invasive Blood Glucose Monitoring Using Machine Learning and Optical Approaches: A Systematic Literature Review and a Proposed Framework for Reproducible and Clinically Reliable Research
Source: Sensors (Basel). 2026 Oct 16;26(20):6572. doi: 10.3390/s26206572 (PMC13708778; doi:10.3390/s26206572)
Supplement: Supplementary file 1 [file sensors-26-06572-s001.zip › Supplementary_Table_S1_Quality_Rubric.pdf]

Supplementary Table S1. Quality-Assessment Rubric

Each study that passed title and abstract screening was scored on its full text across seven domains, for a maximum of 100 points. The table below lists each domain, its maximum score, and how points were assigned at every level.

Domain	Maximum Points	How Points Were Given
1. Publication venue quality	10	Journal = maximum 10; conference proceedings = maximum 8; questionable or unverifiable venue = 0
2. Instrumentation and data collection	30	Own hardware and own data from real subjects = 30; existing hardware but own real data = 18; public or secondary dataset only = 6; no real data collected = 0
3. Study population	5	Population clearly reported (sample size, diabetic status, demographics) = 5; partial reporting = 3; not reported = 0
4. Reference standard and performance reporting	20	Validated glucometer and clinically-oriented metrics (MARD, Clarke or Parkes Error Grid) = 20; glucometer and point-error metrics only (MAE, RMSE, R^2) = 12; unclear reference standard = 5; none reported = 0
5. Validation methodology	15	Subject-wise cross-validation and independent external validation = 15; subject-wise cross-validation only = 9; unclear or sample-wise splitting = 4; not described = 0
6. Machine-learning / deep-learning rigor	10	Several models compared, with a clear description of training and testing = 10; one model, basic description = 5; unclear = 0
7. Confounder transparency	10	Two or more confounders reported (e.g., skin pigmentation, age, temperature, motion) = 10; one confounder reported = 5; none reported = 0
**Total**	**100**	**Studies scoring 63 or more were retained for the review**

Table S1. Seven-domain quality-assessment rubric used for full-text screening (maximum 100 points).

Justification of the 63-point inclusion threshold

A difference of a few points between two studies represents ordinary variation and does not, by itself, indicate that one study is meaningfully better than another. A single stated cut-off was nevertheless required to decide which studies to retain. This was set at 63 out of 100.

This specific value was not chosen in advance. Nine studies scored exactly 63. A higher cut-off, such as 64, would have required excluding some of these nine studies while retaining others with an identical score, with no methodological basis for preferring one over another. Setting the threshold at 63 avoided this problem, since every study scoring 63 or above was retained as a complete group. Studies scoring below 63 were excluded from the review.

Both authors together re-verified the whole process and after a thorough discussion and analysis, 37 studies were finally selected to be a part of our systematic literature review.
